# Methylamine-assisted growth of uniaxial-oriented perovskite thin films with millimeter-sized grains

**DOI:** 10.1038/s41467-020-19199-6

**Published:** 2020-11-06

**Authors:** Haochen Fan, Fengzhu Li, Pengcheng Wang, Zhenkun Gu, Jin-Hua Huang, Ke-Jian Jiang, Bo Guan, Lian-Ming Yang, Xueqin Zhou, YanLin Song

**Affiliations:** 1 Key Laboratory of Green Printing, Institute of Chemistry, Chinese Academy of Sciences, 100190 Beijing, P.R. China; 2 School of Chemical Engineering and Technology, Tianjin University, 300072 Tianjin, P.R. China

**Keywords:** Solar cells, Solar cells

## Abstract

Defects from grain interiors and boundaries of perovskite films cause significant nonradiative recombination energy loss, and thus perovskite films with controlled crystallinity and large grains is critical for improvement of both photovoltaic performance and stability for perovskite-based solar cells. Here, a methylamine (MA^0^) gas-assisted crystallization method is developed for fabrication of methylammonium lead iodide (MAPbI_3_) perovskite films. In the process, the perovskite film is formed via controlled release of MA0 gas molecules from a liquid intermediate phase MAPbI_3_·xMA^0^. The resulting perovskite film comprises millimeter-sized grains with (110)-uniaxial crystallographic orientation, exhibiting much low trap density, long carrier lifetime, and excellent environmental stability. The corresponding perovskite solar cell exhibits a power conversion efficiency (PCE) of ~ 21.36%, which is among the highest reported for MAPbI_3_-based devices. This method provides important progress towards the fabrication of high-quality perovskite thin films for low-cost, highly efficient and stable perovskite solar cells.

## Abstract (web summary)

Suppressing grain boundaries and defects in perovskite solar cells remains a quest to address the efficiency and stability issues. Here, the authors use methylamine gas for assisting the growth of uniaxial-oriented perovskite thin films with millimeter-sized grains.

## Introduction

Organic–inorganic hybrid perovskites—such as MAPbX_3_ (X = I^−^, Br^−^, or Cl^−^)—have attracted increasing attention as light-harvesting materials in low-cost and high-efficiency photovoltaic devices due to their solution processability and excellent optical and electrical properties, such as strong light absorption, long electron–hole diffusion length, and low exciton-binding energy^1–12^. As an active layer, the quality of the perovskite films (coverage, grain size, and crystal orientation) is strongly related to the device performance and stability^13–16^. It is generally believed that the primary energy loss in perovskite solar cells is ascribed to the nonradiative recombination of carriers due to trap states at grain boundaries, surfaces, and point defects within the crystallites^17–24^. The high coverage of perovskite film ensures adequate absorption and avoids possible short circuit, and the crystal domains with large grain size and highly orientation are believed to reduce the density of grain boundaries and promote the efficient transport of electrons and holes to their corresponding electrodes. Concurrently, these defects such as the grain boundaries may cause water molecule diffusion and ion migration, deteriorating the device stability^25–27^. Therefore, a perovskite film with controlled crystallinity and large grains is highly desirable for efficient and stable solar cells.

In order to improve the quality of the perovskite films, some effective methods have been reported by using various solution processing techniques, such as solvent engineering^28,29^, solvent annealing^30^, hot casting^31^, intramolecular exchange^32,33^, and additive-assisted process^27,34,35^. However, the solution-processed perovskite polycrystalline films typically have small grains with sizes of sub-100 nanometers to several micrometers, and the grains in the films usually exhibit various crystallographic orientations, limiting efficient charge transfer in the films^28–35^. By the way, a high-temperature solution-casting method was reported to obtain perovskite film with millimeter-sized domains^31^; however, the domains in the film are not single crystals but rather large-size islands with smaller crystal grains^36,37^. On the other hand, a few research groups have explored the fabrication of single-crystalline perovskite thin films to remove the above-mentioned imperfections in the polycrystalline films^38–40^. However, it is a great challenge to directly grow a single-crystalline layer on a desired substrate with both large lateral dimensions and thin film thickness (<1 μm), which is desirable for high-efficiency perovskite solar cells^38–40^. In 2015, Zhou et al. reported a methylamine (MA^0^) induced defect-healing (MIDH) method for the processing of highly uniform and crystalline MAPbI_3_ perovskite films from a liquid intermediate MAPbI_3_·*x*MA^0^ ^41^. Since then, the MIDH method has been extensively employed for improving the quality of perovskite films^41–55^. In these reports, the grain sizes in the perovskite films are usually limited to 100–200 nm due to the rapid growth of the perovskite films from the rapid MA^0^ self-degassing process.

In this work, a modified MIDH method is developed for the fabrication of MAPbI_3_ perovskite films, where the nucleation and crystallization of the perovskite can be adjusted by controlled release of MA^0^ gas from the liquid intermediate. The resulting perovskite film presents (110)-uniaxial crystallographic orientation with millimeter-sized grains, exhibiting much low trap density and long carrier lifetime. The perovskite solar cells have been fabricated, yielding a stabilized power conversion efficiency (PCE) of ~21.36% with negligible hysteresis and excellent environmental stability.

## Results

### Methylamine gas-assisted growth of perovskite film

**Fig. 1:**
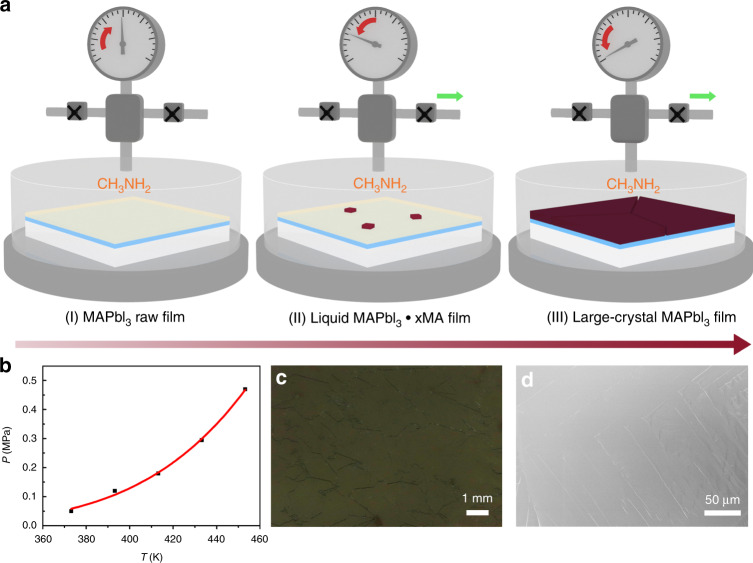
Diagram of the methylamine gas-assisted crystallization method for the deposition of the large-grain MAPbI_3_ perovskite films. **a** The steps of the method: (I) formation of a liquid CH_3_NH_3_PbI_3_·*x*MA intermediate film; (II) controlled growth of the nuclei; and (III) controlled growth of the perovskite film. **b** Equilibrium partial pressure of the MA^0^ gas as a function of temperature for the MAPbI_3_ perovskite crystallization. **c** Photographic image and **d** top-view SEM image of the large-grain MAPbI_3_ perovskite film.

We prepared a methylammonium lead iodide (MAPbI_3_) perovskite film by combining solution deposition and methylamine-assisted method. The basic steps are illustrated schematically in Fig. 1a. First, a pristine MAPbI_3_ film is deposited on a mesoporous TiO_2_ substrate via simple one-step solution method^1–3^. After drying, the perovskite film is loaded face up in a closed chamber in which reagent mixture of methylammonium chloride and sodium hydroxide is enclosed. At an elevated temperature, MA^0^ gas is created inside, and a liquid intermediate MAPbI_3_·*x*MA^0^ is formed^41–43^. Generally, when a raw MAPbI_3_ thin film is exposed to CH_3_NH_2_ (MA^0^) gas source at room temperature (RT), a liquid intermediate MAPbI_3_·*x*MA^0^ is quickly formed through uptake of the MA^0^ gas molecules, where the equilibrium is built, as shown below:

1 $${\mathrm{MAPbl}}_3 \cdot x{\mathrm{MA}}^0\left( 1 \right) \rightleftharpoons {\mathrm{MAPbl}}_3\left( {\mathrm{s}} \right) + x{\mathrm{MA}}^0\left( {\mathrm{g}} \right)$$

The liquid phase is metastable only under a certain partial pressure of MA^0^ gas (*P*_MA_), and upon removal of the MA^0^ gas atmosphere, Eq. 1 will move to the right quickly due to the decreased *P*_MA_ (close to 0). This results in rapid release of the MA^0^ molecules from the liquid phase (<1 s), inducing supersaturation and thus the formation of massive nuclei. The process is uncontrollable and dominated by the nucleation process, thus leading to formation of fine grained (~100 nm in size) thin films.

In this report, the crystallization of MAPbI_3_ is processed at a certain temperature in a closed system in MA atmosphere with a controlled *P*_MA_. The intervals of the *P*_MA_ for the crystallization are determined at a certain temperature ranging from 100 to 180 °C. (Supplementary Table 1; for details, see “Methods”), and a mid-interval value is roughly assigned as the equilibrium *P*_MA_ at the temperature (Fig. 1b). The enthalpy is calculated to be ${\Delta} _rH_m^\theta$ = 150.14 kJ mol^−1^ based on the van’t Hoff equation assuming that *x* is 4 in the intermediate (Supplementary Fig. 1). In this case, the supersaturation could be greatly reduced by slow release of MA molecules from the liquid intermediate (by altering the *P*_MA_ in the interval), leading to low nucleation density and allowing more time for the growth of the large grains in the interval. This process is dominated by the slow growth crystallization process, significantly different from the fast nucleation-driven process reported previously^41^. The film-forming process is similar to the previous report, where the MAPbI_3_ film with tens of micron-sized grains was formed from the liquid phase under a constant MA atmosphere by tuning the annealing temperature^43^.

For clearly exemplifying the unique process, a MAPbI_3_ perovskite film was intentionally prepared from the liquid phase MAPbI_3_·*x*MA^0^ on a porous TiO_2_ film at 120 °C (the *P*_MA_ interval: 0.144–0.096 Mpa), where the MA gas was slowly released from 0.144 to 0.11 MPa, and then rapidly discharged. The optical microscopic image of the resultant perovskite film was recorded as shown in Supplementary Fig. 2. In the image, one large crystal (~1 mm in size) with a regular shape (square) is observed, which is surrounded by a large number of small grains with size about 100 μm. The result may indicate that low nucleation density can be realized in the crystallization through the slow release of the MA^0^ gas.

In our case, we observed that the grain size was strongly dependent on the release rate of MA gas from the liquid phase (discussed below), that is, the degree of the supersaturation. Thus we speculate that homogeneous nucleation could occur in the system. The slow release of MA gas from the liquid phase may favor for the formation of low nucleation sites and also allow more time for the growth of large grains. It should be noted that nonclassical nucleation mechanisms may also be involved here, and further investigation is needed to elucidate the underlying mechanisms.

Figure 1c shows a typical photographic image of the perovskite film, prepared at 120 °C with a 30-s MA^0^ degassing time. The film has uniformly packed grains with grain sizes 1–5 mm, which is about four orders of magnitude larger than that in the MAPbI_3_ film processed by the MIDH method (Supplementary Fig. 3b)^41^. In the following studies, we name the 30-s sample and the MIDH sample as large-grain film and small-grain film, respectively. Figure 1d shows the scanning electron microscopic (SEM) image of the large-grain perovskite film. The top-view images of the MAPbI_3_ perovskite films with low and high magnification are presented in Supplementary Fig. 3c, d, respectively, demonstrating the formation of a smooth, dense, and uniform perovskite layer by the current method.

**Fig. 2:**
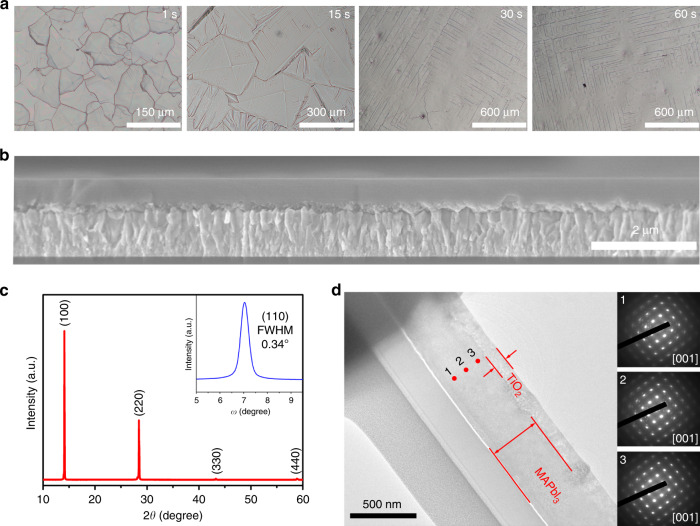
The characterization of MAPbI_3_ perovskite films. **a** Optical microscopic images of the MAPbI_3_ perovskite films prepared from the liquid intermediate with different degassing times (1, 15, 30, and 60 s) in the internal. **b** Cross-sectional scanning electron microscopic (SEM) image of the large-grain MAPbI_3_ perovskite film. **c** X-ray diffraction (XRD) pattern and rocking curve (inset) of the large-grain MAPbI_3_ perovskite film. **d** Cross-sectional transmission electron microscopic (TEM) image and selected area electron diffractions (SAED) taken from the different locations (labeled 1–3) from bottom to top in the perovskite film.

Figure 2a shows optical microscopic images of the perovskite films, evolved from the liquid intermediate with different MA^0^ degassing times within the internals of the crystallization. It is clear that the grain size in the films is strongly dependent on the time. Upon rapid degassing (~1 s) at 120 °C, the grain size is only tens of micrometer, while the size is greatly increased to millimeters when the degassing time is extended to 30 s. With further extending the degassing time (60 s), the grain size seems unchanged. Figure 2b shows the cross-sectional SEM image of the large-grain film on a TiO_2_-coated substrate. The perovskite film is smooth and flat with no discernable grain boundaries over the entire measure range, consistent with the top-view SEM images (Supplementary Fig. 3c, d). In addition, we prepared the large-grain films at different temperatures with 30 s degassing time, and the top-view SEM and optical microscope images are shown in Supplementary Figs. 4 and 5. The grain sizes were comparable for all the large-grain films, indicating that the grains have already grown up within the time at the different temperatures. Supplementary Fig. 6 presents the optical microscopic images of the MAPbI_3_ films prepared at low temperatures below 373 K. In addition, the atom force microscope (AFM) was employed to characterize the morphologies of the small-grain film and different large-grain films with different degassing time (1, 15, 30, and 60 s). The root mean square roughness value of the different large grain films are comparable, with 0.67 nm for 1 s, 0.73 nm for 15 s, 0.61 nm for 30 s, and 0.76 nm for 60 s, respectively, all of which are lower than that (2.1 nm) for the small-grain film (Supplementary Fig. 7). However, large roughnesses are observed in the regions of the texture (3.1 nm, corresponding to the square line observed in the SEM) and the grain boundary (42 nm), as shown in Supplementary Fig. 8. Kelvin probe force microscopy (KPFM) mapping was also recorded across two adjacent grains (Supplementary Fig. 9).

The ultraviolet–visible (UV–Vis) spectra of the large-grain film and the pristine perovskite film are shown in Supplementary Fig. 10a. Both the films show almost the same absorption edges, and the large-grain film presents stronger absorption at the full light absorption range when compared with the pristine film. The steady-state photoluminescence (PL) spectra show that the emission peak intensity for the large-grain film is nearly two orders of magnitude higher than that of the pristine film, indicative of reduced surface defect and enhanced crystallinity in the large-grain film (Supplementary Fig. 10b). The inset in Supplementary Fig. 10b shows a 12-nm blue shift of the PL peak for the large-grain perovskite film.

### Properties of the perovskite film

The X-ray diffraction (XRD) pattern of the large-grain film in Fig. 2c exhibits intense signals only in the <110> crystalline directions, indicating that the exposed face is {110} crystal plane in the film. The XRD pattern of the powders (scratched from the film) matches a tetragonal crystal structure (Supplementary Fig. 11), which is the most stable phase at RT^56^. In addition, a rocking curve, which is the most reliable measure of the average orientation of a particular crystallographic phase, was measured for the large-grain film, exhibiting a small full width of 0.34° at half maximum (FWHM) for the {110} plane. The FWHM value is one order of magnitude lower than those for the polycrystalline perovskite films with micrometer-sized grains^21,57^, and is close to the value (0.33°) for the single-crystal perovskite thin film reported previously^39^, further demonstrating that the grain has a sharp preferred orientation. In order to get more information of the grain orientation, one large grain cut from the film was measured by two-dimensional detector. As shown in Supplementary Fig. 12, only two visible diffraction points (not arcs) and the point of beam are observed, further indicating that the large grain has a strong preferred orientation. Moreover, the polarized optical microscopic images of the large-grain film were recorded, as shown in Supplementary Fig. 13, where the extinction or transmission state was observed across one large grain from the film when measured by changing the angle of polarizers.

To verify the uniformity of the crystallographic orientation in radial direction in the large-grain film, selected area electron diffraction (SAED) was performed on the cross-section specimen, prepared with the focused ion beam (FIB) along the growth striation on the surface. As shown in Fig. 2d, three SAED patterns, taken at the top, middle, and bottom locations of the specimen, are consistent with the single-crystal tetragonal phase with the same zone axis [001], confirming a single-crystalline structure in the area from the bottom to top.

**Fig. 3:**
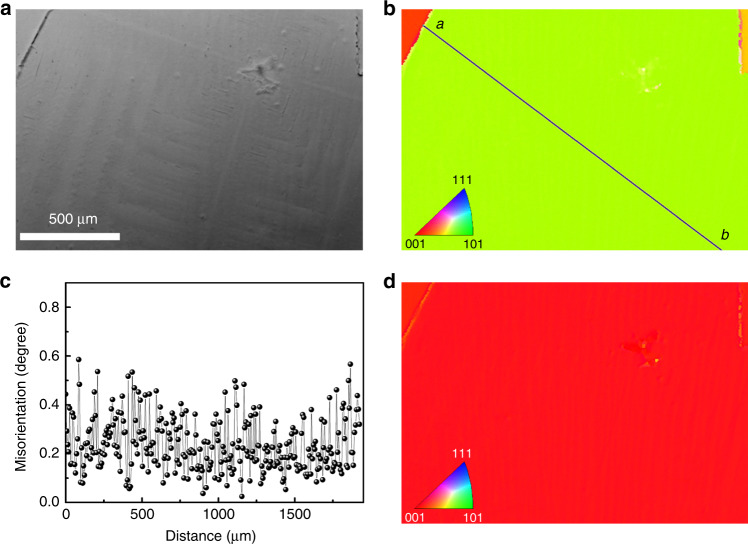
Electron backscatter diffraction (EBSD) of the large-grain MAPbI_3_ film. **a** Backscattered electron (BSE) image. **b** *X* axis inverse pole figure (IPF) (parallel to the sample surface). **c** Point-to-point misorientation profile along the black line from *a* to *b* in **b**. **d** *Z* axis inverse pole figure (normal to the sample surface). The scale bar in **a** also applies to **b**, **d**.

To inspect the phase purity and the quality over a large scale for the large-grain perovskite film, a SEM-based electron backscatter diffraction (EBSD) technique was employed^58^. In the experiment, a focused electron beam illuminates at an angle of 70° to the surface of the film, and the diffraction pattern (Kikuchi patterns) is then transformed into the crystallographic orientation map through statistical analysis. As shown in Fig. 3a, an area of 1.75 × 1.21 mm^2^ was randomly selected from the film for the experiment. In the *X* axis inverse pole figure (Fig. 3b), each individual grain presents nearly the same color, indicating the same crystallographic orientation. A point-to-point misorientation difference, measured along the diagonal line of one grain, is measured to be <0.6° (Fig. 3c), indicating perfect crystal orientation within the grain^59^. Most interestingly, all the grains in the image present uniform color in the inverse pole figure *Z* direction, normal to the sample surface (Fig. 3d), implying that all crystallites are very well oriented with the {110} planes, parallel to the substrate. The results are in line with the XRD measurement. This uniaxial orientation is highly desirable for efficient charge transport in the perovskite-based optoelectronic devices. It should be noted that, in the traditional solution-processed perovskite films, various solid-state plumbate intermediates are inevitably generated during the growth of the perovskites, and the resulting films usually present random crystallographic orientations^60,61^. Here the high degree of order obtained could be ascribed to only phase-pure MAPbI_3_ crystallization from the solvent-free liquid phase, and a similar result was observed for the MAPbI_3_ film crystallized from an ionic liquid^62^.

We also prepared the perovskite film at 120 °C with a short degassing time (~2 s). As shown in Supplementary Fig. 14, the fast degassing process resulted in the formation of relatively small grains with a broad size distribution (50–150 µm) in the film. While each individual grain shows a uniform color in the *X* axis inverse pole figure, indicating single orientation, the image present non-uniform color distribution in the inverse pole figure *Z* direction, implying the film lacking of complete orientation. The result indicates that a slow crystallization rate is necessary for the growth of the MAPbI_3_ film with both uniaxial orientation and large grains. We also tried to measure the EBSD mapping of the small-grain film, and the mapping is shown in Supplementary Fig. 15, where the indexing success rate is only 0.57% with little effective information about the crystal orientation. Under the identical conditions, the EBSD mapping of the large-grain film can be measured, as shown in Supplementary Fig. 16. The difference could be explained as follows: for small-grain film, the scanning range is relatively small, and the probe beam current is the same as the same dwell time on the sample surface. In this case, the sample will receive more electrons per unit area at the same time, causing larger damage on the small-grain film as compared with the large-grain film. In addition, we extended the method for the fabrication of large-grain MAPbBr_3_ film, and the results are shown in Supplementary Figs. 17 and 18.

**Fig. 4:**
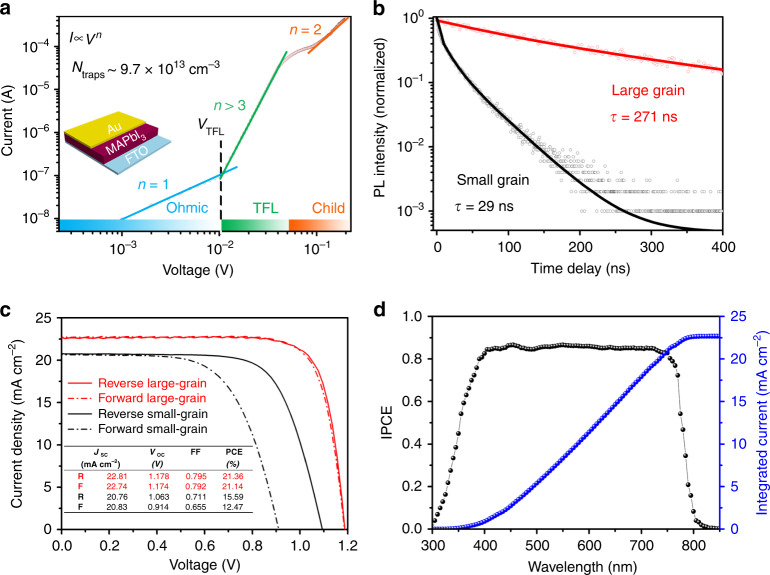
Characterization of the large-grain MAPbI_3_ perovskite films. **a** Current–voltage (*I*–*V*) trace of the large-grain MAPbI_3_ film, showing Ohmic, TFL, and Child region. The onset voltage of the TFL region is ~0.011 V. **b** Time-resolved photoluminescence (TRPL) decay measurements of the large- and small-grain MAPbI_3_ films. **c** Characteristic *I*–*V* curves of the large- and small-grain devices. Solid lines and dashed lines indicate the reverse- and forward-bias scan directions, respectively. **d** The IPCE spectrum of the large-grain device and the integrated short-circuit current density (*J*_SC_) versus wavelength.

To evaluate the trap-state density (*N*_t_), the large-grain MAPbI_3_ film was inserted between fluorine-doped tin oxide (FTO) and gold electrodes, and the evolution of the space-charge-limited current at different biases was recorded. The trap density (*N*_t_) is estimated based on the equation: *N*_t_ = 2*ε*_0_*ε*_r_*V*_TFL_/*eL*^2^, where *ε*_0_ and *ε*_r_ are the absolute and relative dielectric constants, respectively, *e* is the elemental charge, *V*_TFL_ is the onset voltage, and *L* is the thickness of the film. As shown in Fig. 4a, the large-grain film exhibits a remarkably low trap density *N*_t_ = ~9.7 × 10^13^ cm^−3^, which is more than two orders of magnitude lower than that (~1.6 × 10^16^) for the small-grain MAPbI_3_ film (Supplementary Fig. 19). The significantly reduced *N*_t_ can originate from the high crystallinity and fewer grain boundaries in the large-grain perovskite film^19,35^. To further study the charge dynamics, the time-resolved photoluminescence (TRPL) spectroscopies were performed for both the large- and small-grain MAPbI_3_ films (Fig. 4b). Fitting the TRPL data with a stretched exponential function, average carrier lifetimes of 271 and 27.8 ns are extracted for the large-grain film and the small-grain film, respectively. The longer lifetime of the large-grain film is indicative of slow recombination due to the reduced defect sites in the large-grain perovskite film, which is consistent with the high steady-state PL intensity. The high PL intensity and long PL decay lifetime illustrate that the nonradiative recombination of photo-generated carriers is significantly reduced due to the high crystallinity and low density of grain boundaries in the large-grain perovskite film.

### Solar cell performance

To evaluate the photovoltaic metrics of the large-grain MAPbI_3_ film, a device configuration of FTO/compact TiO_2_/meso-TiO_2_/perovskite/spiro-OMeTAD/Au was constructed to measure photocurrent density versus voltage (*J*–*V*) curve and incident photon-to-current conversion efficiency (IPCE). For comparison, the small-grain MAPbI_3_ film was employed with the same device configuration. The statistical data on the PV metrics for both the devices are listed in Supplementary Fig. 20. The *J*–*V* curves of champion devices are presented in Fig. 4c with the figures of the merit in the inset. For a reverse scan, the large-grain device shows a PCE of 21.36% with short-circuit current voltage (*J*_SC_) of 22.81 mA cm^−2^, open-circuit voltage (*V*_OC_) of 1.178 V, and fill factor (FF) of 0.795, whereas the small-grain device delivers a lower PCE of 15.69% with *J*_SC_ of 20.76 mA cm^−2^, *V*_OC_ of 1.063 V, and FF of 0.711. For a forward scan, the former shows negligible hysteresis with a PCE of 21.14%, while the latter exhibits large hysteresis with a PCE of 12.47%. The low hysteresis can be ascribed to the low defect sites in the perovskite film with large grains and high crystallinity, which effectively prevent ion mobility and thus charge accumulation at the perovskite surface^17–19^. Figure 4d shows high IPCE values over the spectral range from 400 to 800 nm for the large-grain device, which is in accordance with the optical absorption profile. The integrated *J*_SC_ (22.67 mA cm^−2^) from the IPCE is in good agreement with the *J*–*V* measurement. A stabilized PCE of ~21.36% is recorded for the large-grain device by holding the voltage at the maximum power point (Supplementary Fig. 21), which is among the highest values for the single-cation/anion perovskites^28–35^. The best device has been certified by a third party (Supplementary Fig. 22), exhibiting 21.29% under the reverse scan direction (1.2 to −0.1 V) and 20.12% under the forward scan direction (−0.1 to 1.2 V) under AM 1.5 G full-sun illumination (100 mW cm^−2^). In addition, the *I*–*V* curves of the different grain-size-based devices (time is 1, 15, 30, or 60 s for the crystallization of the MAPbI_3_ films) were prepared, as shown in Supplementary Fig. 23. We observed that the device-1 s showed relatively poor conversion efficiency (~19%), as compared with the other three devices, all of them have comparable efficiencies (~21%). The low efficiency for the device-1 s may be ascribed to the small grain and relatively poor orientation in the film, as evidenced by the relatively low values of *V*_OC_ and FF.

**Fig. 5:**
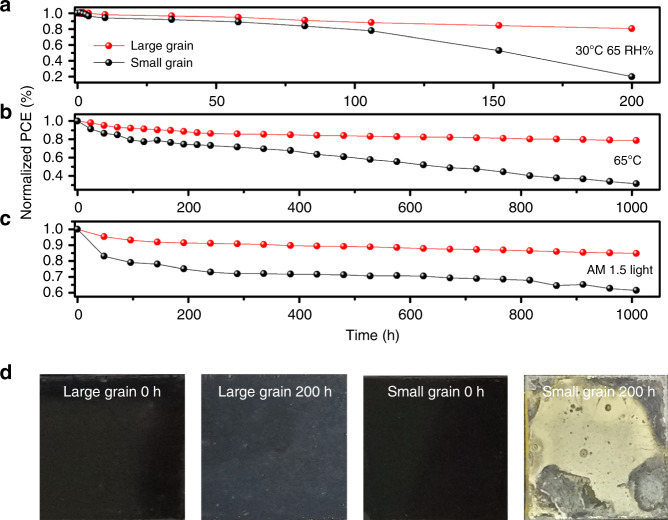
Stability performance measurements. **a**–**c** Stability assessment of non-encapsulated perovskite solar cells with the large-grain and the small-grain perovskite films. Humidity stability at 65 RH% and 30 °C in air (**a**), heat stability at 65 °C in nitrogen environment (**b**), and light stability testing under full-spectrum sunlight at room temperature in nitrogen environment (**c**). **d** Photographs of the large- and small-grain perovskite films before and after exposure to humidity of 65% for 200 h at room temperature in air (the size of the films is 20 × 20 mm^2^).

The deficiency of long-term stability of perovskite materials has become one of the greatest barriers to the commercialization, and the degradation is generally initialized from the defect sites at surfaces and grain boundaries, which are more reactive toward moisture. For poly-crystalline perovskite films with sub-micrometer-sized grains, many organic molecules have been deposited on the film to passivate the defects during the past years^10–12^. In our cases, the large-grain perovskite films without the post-treatment were constructed in the solar cells for stability test. The environmental stability of the devices without encapsulation was tested at 65 RH% at 30 °C in air, at 65 °C in nitrogen environment, and under thermal stressing and full-spectrum sunlight irradiation, respectively. The results are shown in Fig. 5a–c. At 65 RH% and 30 °C, the device employing the large-grain perovskite film shows greatly improved stability in comparison with that of the small-grain device. The large-grain device retains over 80% of its initial PCE after 200 h, whereas the control device decays to <20% of the original performance. In addition, the XRD patterns of the large- and small-grain films were recorded before and after exposure to humidity of 65% for 200 h at RT in air, and the results are shown in Supplementary Fig. 24. After the 200-h aging, a set of peaks of PbI_2_ appeared with high intensity for the small-grain film, showing poor stability. In contrast, the large-grain film showed greater stability, showing very weak peaks from PbI_2_ and the hydrate intermediate under the same aging condition. The enhanced humidity stability is likely to be attributed to the intrinsic properties such as uniaxially oriented grains and reduced grain boundaries, which can effectively inhibit the permeation of moisture in the large-grain perovskite film^25^. As shown in Fig. 5d, the large-grain film did not change markedly in color over 200 h, showing excellent stability, whereas the small-grain film turned yellow under the same conditions, because of fast decomposition of MAPbI_3_ with water, and forming PbI_2_ in the small-grain film. Likewise, the enhanced stability in the large-grain device can also be observed under thermal stressing and full-spectrum sunlight irradiation, as shown in Fig. 5b, c. The large-grain devices retain around 80 and 85% of their initial PCEs, respectively, under thermal stressing at 65 °C and under the illumination after 1000 h, whereas the control devices decay to about 30 and 60% of the original PCEs, respectively, under the identical conditions. The high stability under the illumination and the heat could be ascribed to effective suppression of ion migration in the large-grain perovskite film with high crystallinity and reduced grain boundaries^26,27^. The significantly improved stability as well as high efficiency indicate the great potential of the large-grain perovskite solar cell for industrial applications.

## Discussion

In summary, the methylamine gas-assisted crystallization method was developed for the fabrication of high-quality perovskite films. In the process, the perovskite film is formed from a liquid intermediate phase MAPbI_3_·*x*MA^0^ via controlled release of MA^0^ gas molecules. By the method, we obtained a uniaxial-oriented perovskite films with millimeter-sized single-crystal grains, which are comprehensively evaluated by using XRD, XRD-rocking curve, and EBSD techniques for the first time. The large-grain perovskite film possesses a low trap-state density and long charge carrier lifetime, which are comparable to those of the single-crystal thin film. The resulting perovskite solar cells exhibited both high efficiency and excellent environmental stability. This represents a major advance in the fabrication of high-quality perovskite films. On the basis of our results, we believe that the uniaxial-oriented large-grain perovskite films will find widespread application in low-cost, high-performance, and stable perovskite optoelectronics devices.

## Methods

### Materials

Lead (II) iodide (99.999%) was purchased from Sigma Aldrich. Methylammonium chloride, methylammonium bromide (>99.5%), methylammonium iodide (>99.5%), and Spiro-OMeTAD were purchased from Xi’an Polymer Light Technology in China.

### Fabrication of TiO_2_ electrode

FTO glasses (TECA7, 6–8 ohm sq^−1^, NSG, Japan) were cleaned with deionized water, alcohol, and acetone successively via ultrasonic process and then cleaned with UV ozone for 10 min. A 30-nm TiO_2_ blocking layer was deposited on the FTO by spray pyrolysis at 450 °C from a precursor solution of 20 mM titanium diisopropoxide bis(acetylacetonate) solution. A 150-nm mesoporous TiO_2_ layer was spin-coated on the substrate with a diluted TiO_2_ paste (18NR-T, Dyesol) in ethanol with a weight ratio of 1:6 and then calcined at 500 °C for 30 min in air.

### Methylamine-assisted recrystallization of MAPbI_3_ film

The starting MAPbI_3_ perovskite films were deposited the TiO_2_ electrode using the conventional one-step method, where a 47 wt% PbI_2_:MAI (molar ratio 1:1) solution in *N,N*-dimethylformamide was spin-coated at 177.1 × *g* (~4000 rpm) for 30 s, followed by heat treatment at 100 °C for 10 min. The raw perovskite film was loaded face up in a closed chamber. Before the loading, a beaker with a mixture of methylammonium chloride, sodium hydroxide, and CaO dryer was loaded in advance. After that, the chamber was moved in an oil bath pot at RT, and the air in the chamber was exchanged by N_2_ gas. With the heating by the bath, the pressure rises upon the generation of MA gas. At a certain temperature between 100 to 180 °C, the interval of the MA partial pressure (*P*_MA_) for the crystallization of the MAPbI_3_ were determined using the bisection method according to the morphology change before and after the MA treatment. The pressure can be adjusted by the valve on the chamber. In the interval, a slow MA degassing is required for the film growth. Generally, a degassing time in the internal is within 1 min. After the degassing, N_2_ gas was input, and the chamber was moved out of the oil bath. After being cooled to RT, the sample was taken out. This operation is also suitable for the fabrication of large-grain MAPbBr_3_ film.

### Fabrication of solar cells

On the resulting perovskite film, a HTM was deposited by spin coating a solution, which was prepared by dissolving 72.3 mg (2,29,7,79-tetrakis(*N*, *N*-di-p-methoxyphenylamine)-9,9-spirobifluorene) (spiro-MeOTAD), 28.8 ml 4-tert-butylpyridine, and 17.5 ml of a stock solution of 520 mg ml lithium bis(trifluoromethylsulphonyl)imide in acetonitrile. Finally, 80 nm of gold was thermally evaporated on top of the device to form the back contact.

### Measurement and characterization

The absorption spectra were collected using a UV/Vis spectrometer (SHIMADZU, UV-1800 UV/Vis Spectrophotometer) in the wavelength range of 300–900 nm. Steady-state PL was measured using Edinburgh FLS980 system with an excitation at 485 nm. Optical and polarization images of perovskite films were obtained by Nikon LV100ND optical microscope. The powder XRD patterns and rocking curves were measured using a PANalytical Empyrean X-ray powder diffractometer equipped with a 2.2 kW Cu Kα radiation (1.54 Å). The sample of rocking curves were cutting the films with the grain boundaries. The 2D XRD data were obtained at 1W1A Diffuse X-ray Scattering Station, Beijing Synchrotron Radiation Facility (BSRF-1W1A). The AFM and KPFM data were collected on a Bruker Dimension Icon SPM by using doped silicon PFQNE-AL probes (Bruker). The SEM images were taken from a Hitachi S-4800 SEM operated at 5 kV. EBSD patterns were acquired by Symmetry EBSD detector of Oxford Instruments on a Hitachi SU5000 SEM at 20 kV with sample tilted 70° and analyzed using the AZtec and Channel 5 softwares.

To obtain the TEM sample, the sample was milled by an FIB (the FEI Helios Nanolab G3 CX). Before milling, 15 nm amorphous carbon was first deposited on the surface of the sample. Then a rectangle area was chosen that was parallel to the crystal grain and 700 nm and 1 μm Pt was deposited to protect the sample at 5 kv, 0.17 nA and 30 kv, 80 pA. The milling of the cross-sections used a gallium ion source at 30 kV, 0.79 nA for regular and 5 kV, 7 pA for polishing the cross-section. Finally, a cross-section 70-nm thick was obtained.

Current–voltage (*J–V*) characteristics were recorded by applying an external potential bias to the cell while recording the generated photocurrent with a Keithley model 2400 digital source meter. The light source was a 300 W collimated xenon lamp (Newport) calibrated with the light intensity to 100 mW cm^−2^ under AM 1.5 G solar light conditions by a certified silicon solar cell. The *J–V* curve was recorded by the reverse scans with a rate of 100 mV s^−1^. The active area was determined by metal shadow mask with an aperture of 0.0725 cm^2^. The IPCE for solar cells was performed using a commercial set-up (PV-25 DYE, JASCO). A 300 W Xenon lamp was employed as a light source for the generation of a monochromatic beam. IPCE spectra were recorded using monochromatic light without white light bias. Calibrations were performed with a standard silicon photodiode.

### Device stability testing

The humidity stability for both the unencapsulated perovskite films and the relative devices was conducted in a temperature–humidity chamber (DHS-100, Beijing Zhongkehuanshi Instrument Co. Ltd., China). In the experiment, the temperature is maintained at 30 °C with relative humidity 65%. The photovoltaic performance of the aged devices was measured under ambient conditions.

### The bisection method for the determination of the equilibrium *P*_MA_ range

There are several prerequisites for the determination of the equilibrium *P*_MA_ range:

1. The atmospheric pressure is taken as 0.1 MPa in the calculation.
2. The raw film and chamber must be preheated.
3. This method can roughly measure the methylamine gas interval.

For the measurement of the interval of the equilibrium *P*_MA_, a starting point of the bisection should be determined at first. The lower limit of the interval is initially set to 0, and the upper limit is determined by the following method. At first, adding enough reactants to the chamber, recording the pressure value when the pressure is stable, and then turning valve to let the gas release slowly. The initial value is based on the result of morphological observations by a microscope: if the grains obtained are larger than several tens of microns with texture, the previously recorded pressure value is set as the initial value, otherwise the experiment should be repeated with more amount of reactants to increase *P*_MA_ until the large grains appear.

After determining the initial value, the interval is divided into two sections: the initial value to the half of the initial value, and the half of the initial value to 0. One of the sections is selected, and the gas is slowly released only in the corresponding interval. If large grains appear on the film, it indicates that the equilibrium *P*_MA_ is within this range. If not, it must be in another section. Then the two end points of this interval are reset as the new starting points of the bisection. Repeating the steps for several times, a relatively accurate interval could be determined as to where the equilibrium *P*_MA_ is located.

It should be noted that a special case could remain, as shown in Supplementary Fig. 2, where the equilibrium *P*_MA_ is just near the breakpoint one divided earlier.

It should be emphasized that, after the gas released, the pressure value displayed on the pressure gauge does not indicate the partial pressure of the methylamine gas in the chamber at that time. An example is given below to illustrate the calculation process.

It is assumed that the pressure of methylamine gas in the initial chamber is 0.5 MPa, and the gas is released until the gauge pressure shows 0.3 MPa. At this time, the partial pressure of methylamine gas in the chamber is calculated as follows.

Initial total gas pressure in the chamber:

$$P_{{\rm{ST}}} = 0.5 + 0.1 = 0.6\,{\rm{MPa}}.$$

Final total gas pressure in the chamber:

$$P_{{\rm{ET}}} = 0.3 + 0.1 = 0.4\,{\rm{MPa}}.$$

Initial partial pressure of methylamine gas in the chamber:

$$P_{{\rm{MAS}}} = 0.5\,{\rm{MPa}}.$$

Final partial pressure of methylamine gas in the chamber:

$$P_{{\rm{MA}}} = P_{{\rm{ET}}} \times \frac{{P_{{\rm{MAS}}}}}{{P_{{\rm{ST}}}}} = 0.4 \times \frac{{0.5}}{{0.6}} = 0.33\,{\rm{MPa}}.$$

### Estimate of the enthalpy for the methylamine desorption reaction

In the degassing reaction, the MAPbI_3_ crystallizes from the liquid intermediate MAPbI_3_·*x*MA. Assuming the *x* (≈4) value in the intermediate is not dependent on the MA partial pressure, the degassing reaction can be written as Eq. 2. In the reaction, the enthalpy change was achieved using the well-known van’t Hoff equation ${\int} {{\rm{dln}}K^\theta } = \int {\frac{{{\Delta} _rH_m^\theta }}{{RT^2}}}\, {\rm{d}}T$, where *K*^*θ*^ is the equilibrium constant, *T* the absolute temperature, and *R* the gas constant. In the reaction, the *K*^*θ*^ is determined by the partial equilibrium pressure of the methylamine gas and fugacity coefficients *φ*, as shown in Eq. 3. Supplementary Table 1 lists the intervals of the MA partial pressures for the reaction at different temperatures, and the mid-interval value is roughly assigned as the equilibrium *P*_MA_ at the temperature. *φ* is calculated from Eq. 4, where the acentric factor *ω* is 0.281 according to the reference^63^, and the values of ${\mathrm{log}}(\frac{f}{p})^{(0)}$ and ${\mathrm{log}}(\frac{f}{p})^{(1)}$ at the different temperatures are collected from the reference^64^. Thus the *K*^*θ*^ values at the different temperatures can be calculated according to Eq. 3. Assuming that the enthalpy change is not changed with the temperature, the van’t Hoff equation is given as Eq. 5. The ln*K*^*θ*^ versus 1/*T* is plotted (Supplementary Fig. 1), giving ~150.14 kJ mol^−1^ of the ${\Delta} _rH_m^\theta$.

2 $${\mathrm{MAPbI}}_3\cdot 4{\mathrm{MA}}({\mathrm{l}}) \rightleftharpoons {\mathrm{MAPbI}}_3({\mathrm{s}}) + 4{\mathrm{MA}}({\mathrm{g}});$$

3 $$K^\theta = \left( {\varphi} \right)^4\left( {\frac{P}{{P^\theta }}} \right)^4;$$

4 $${\mathrm{log}}\varphi = {\mathrm{log}}(\frac{f}{p})^{(0)} + \omega {\mathrm{log}}(\frac{f}{p})^{(1)};$$

5 $$\ln K^\theta = - \frac{{{\Delta} _rH_m^\theta }}{{RT}} + C.$$

### Reporting summary

Further information on research design is available in the Nature Research Reporting Summary linked to this article.

## Supplementary information

Supplementary Information

Reporting Summary

## Data Availability

The data that support the findings of this study are available from the corresponding author upon reasonable request.
