# Supplementary material for: Methylamine-assisted growth of uniaxial-oriented perovskite thin films with millimeter-sized grains
Source: Nat Commun. 2020 Nov 6;11:5402. doi: 10.1038/s41467-020-19199-6 (PMC7648077; doi:10.1038/s41467-020-19199-6)
Supplement: Supplementary file 1 — Supplementary Information [file 41467_2020_19199_MOESM1_ESM.pdf]

# Supplementary Information

## Methylamine-Assisted Growth of Uniaxial-Oriented Perovskite Thin Films with Millimeter-Sized Single-Crystal Grains

Haochen Fan[1,2], Fengzhu Li[1], Pengcheng Wang[1], Zhenkun Gu[1], Jin-Hua Huang[1], Xueqin Zhou[2]*, Bo Guan[1], Lian-Ming Yang[1], Ke-Jian Jiang[1]* & Yanlin Song[1]*

**This PDF file includes:**

Supplementary Figure 1-24

Supplementary Table 1

Supplementary Note 1-2

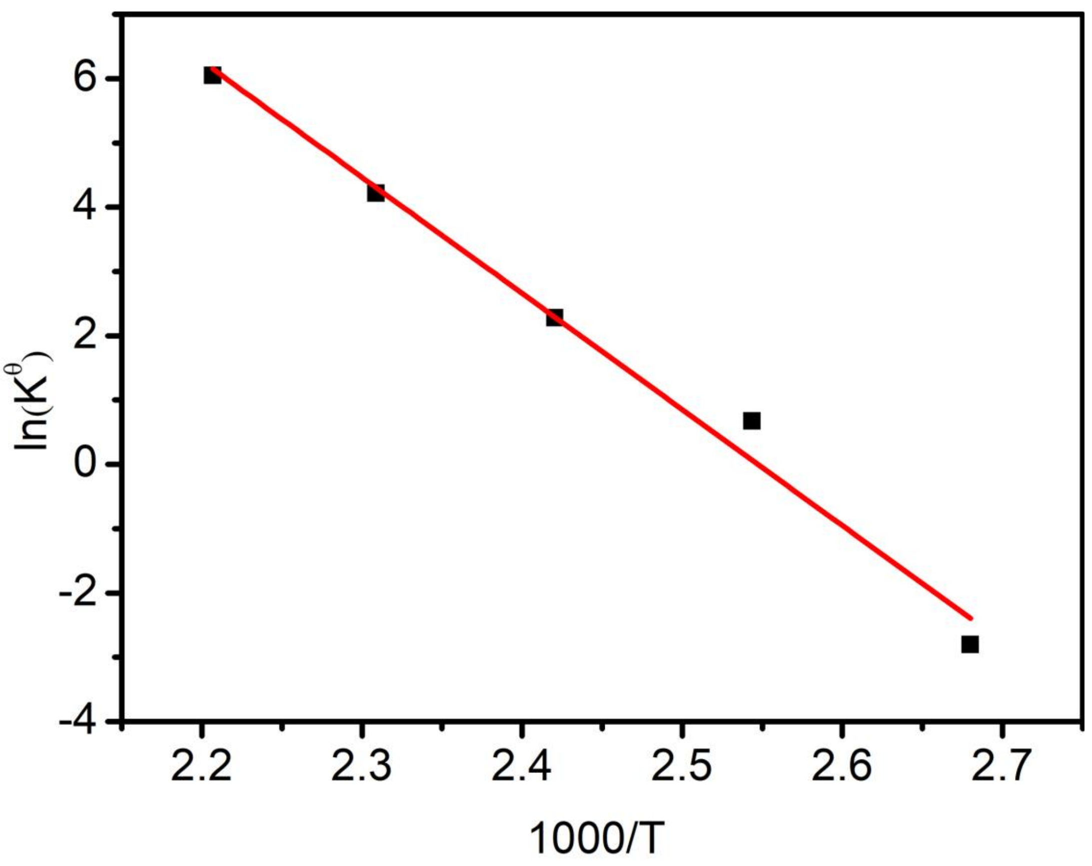


**Supplementary Figure 1| Plot of $\ln K^{\theta}$ versus 1/T for the methylamine desorption reaction.**

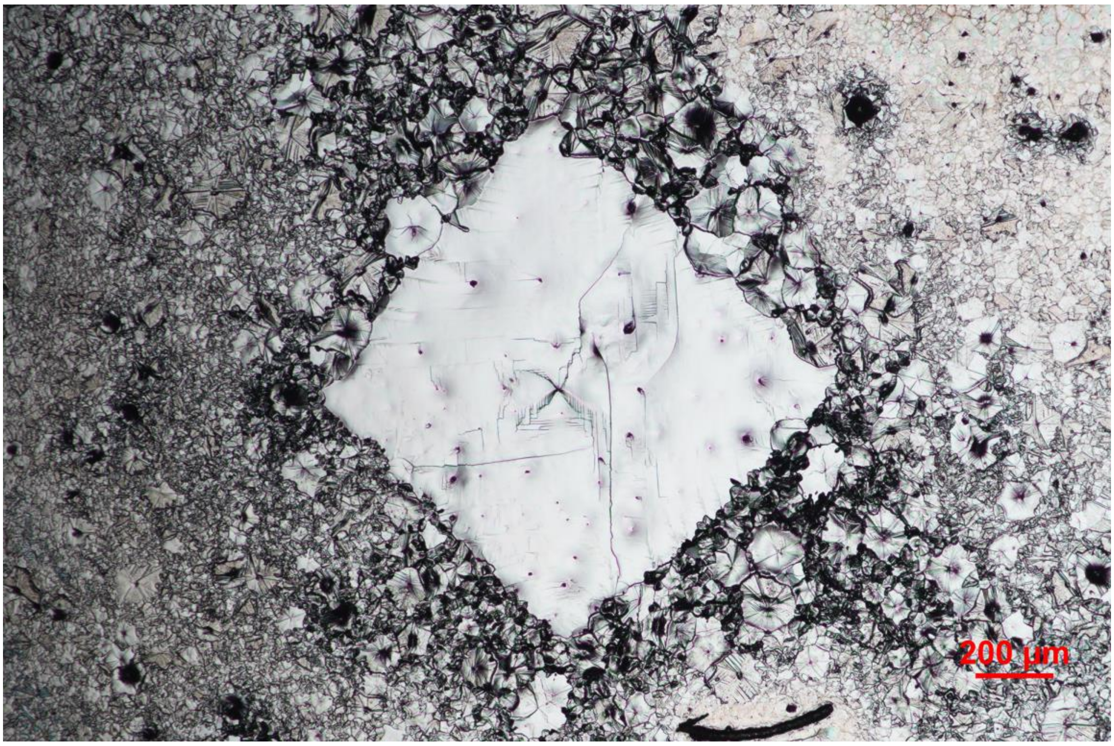


**Supplementary Figure 2| The optical microscope image of the $MAPbI_3$ film formed from the liquid $MAPbI_3 \cdot xMA^0$ at 120 °C with slow release of MA gas from 0.144 to 0.11 MPa, and then rapid discharge at 0.11 MPa.**

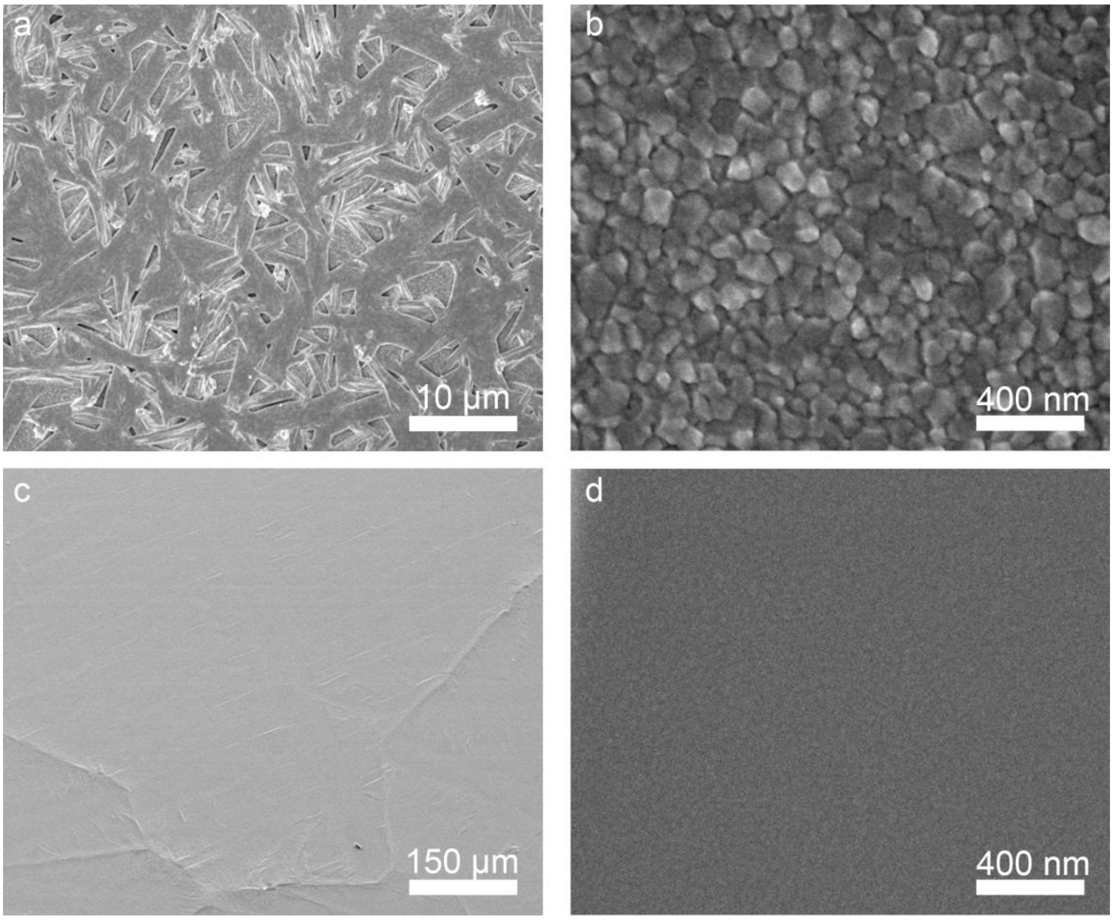


**Supplementary Figure 3| SEM plane view images of $MAPbI_3$ perovskite films.** (**a**) The conventional one-step coated perovskite film. (**b**) The small-grain perovskite film induced by MA gas at RT for (**a**). (**c**), (**d**) Low-magnification SEM (**c**) and higher magnification SEM (**d**) of the large-grain perovskite film.

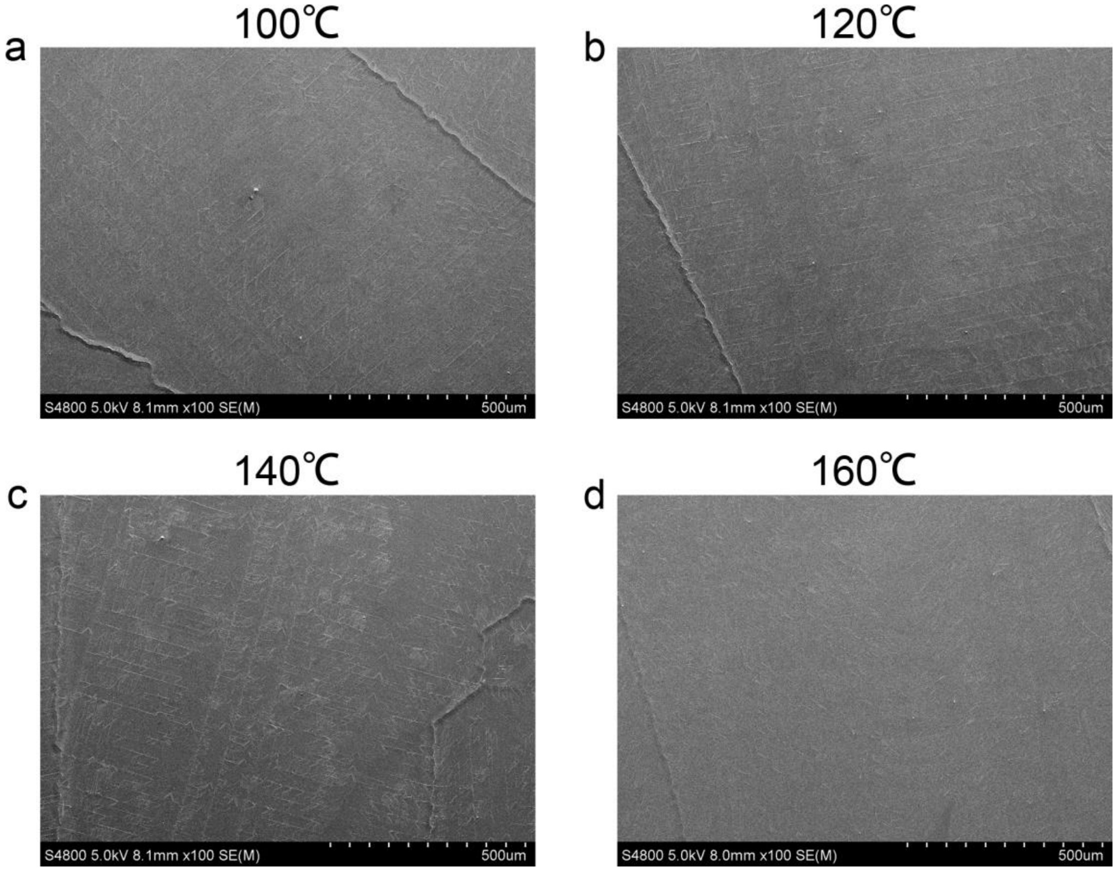


**Supplementary Figure 4| Top-view SEM images of large-grain films prepared at different temperatures.** (**a**) Prepared at 100 ℃. (**b**) Prepared at 120 ℃. (**c**) Prepared at 140 ℃. (**d**) Prepared at 160 ℃.

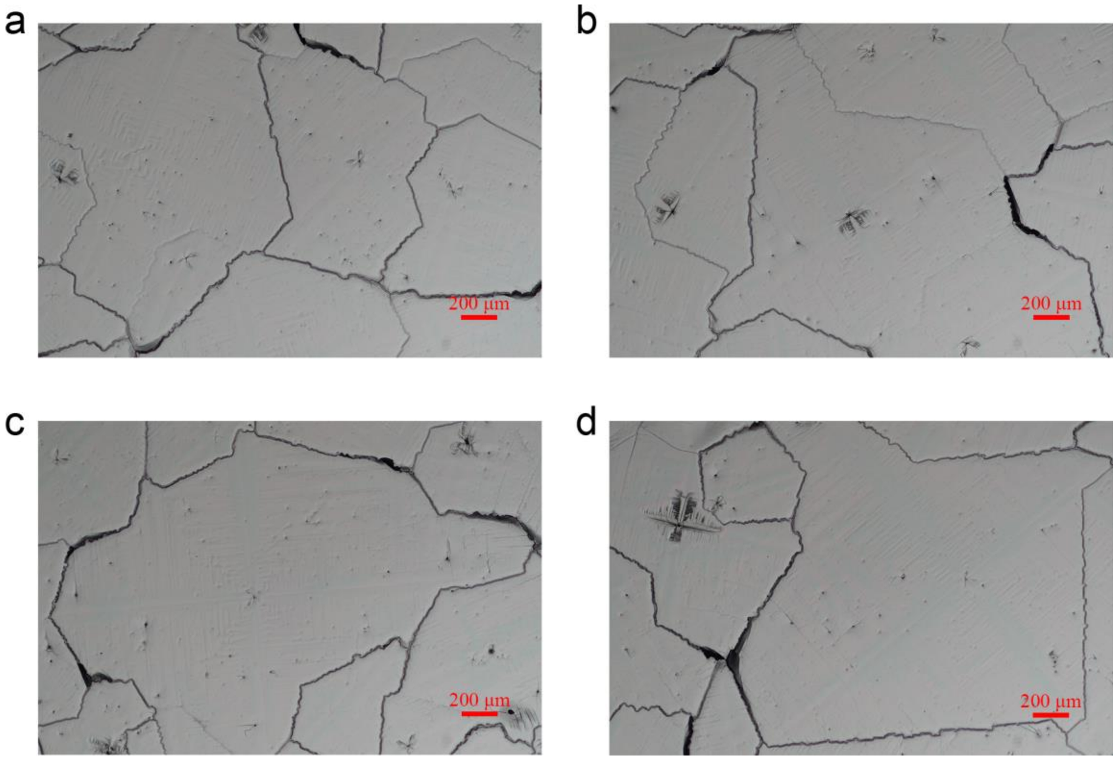


**Supplementary Figure 5| The optical microscope images of the large-grain perovskite films prepared at different temperatures.** (**a**) 100 ℃, (**b**) 120 ℃, (**c**) 140 ℃, and (**d**) 160 ℃.

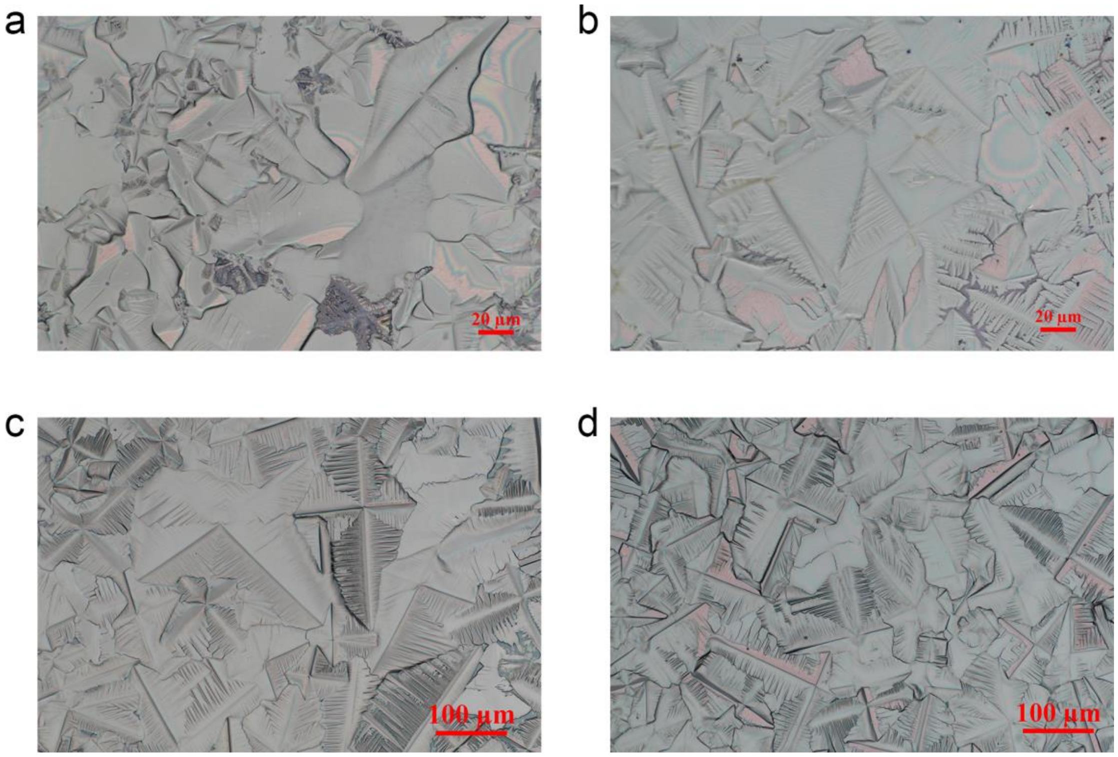


**Supplementary Figure 6| The optical microscope images of the $MAPbI_3$ films prepared at the different temperatures.** (**a**) 25 ℃, (**b**) 40 ℃, (**c**) 60 ℃, and (**d**) 80 ℃.

**Supplementary Note 1: The analysis of perovskite films prepared at temperatures below 373K.**

It is noted that in the experiment, a pressure gauge with measurement range of 0-0.6 MPa and a division value of 0.02 MPa, was employed to measure the gas pressure. When the temperature is lower than 373 K, the $P_{MA}$ of the crystallization section is close to the minimum scale of the pressure gauge. In this case, we repeatedly charged the pressurized nitrogen in the chamber for the controlled release of the MA gas during the crystallization. It is worth nothing that repeated filling with nitrogen will cause certain measurement errors. It is difficult to obtain the crystallization interval at the temperatures below 373 K, and thus we could not determine if the degas is fallen in the internal. In this case, we controlled the degas slowly for the crystallization at the low temperatures (below 373 K). The corresponding optical microscope images are shown in Supplementary Figure 23. As shown in the Supplementary Figure 23, the grain size ranges from tens to hundreds of microns for the films prepared at the different temperatures below 373 K.

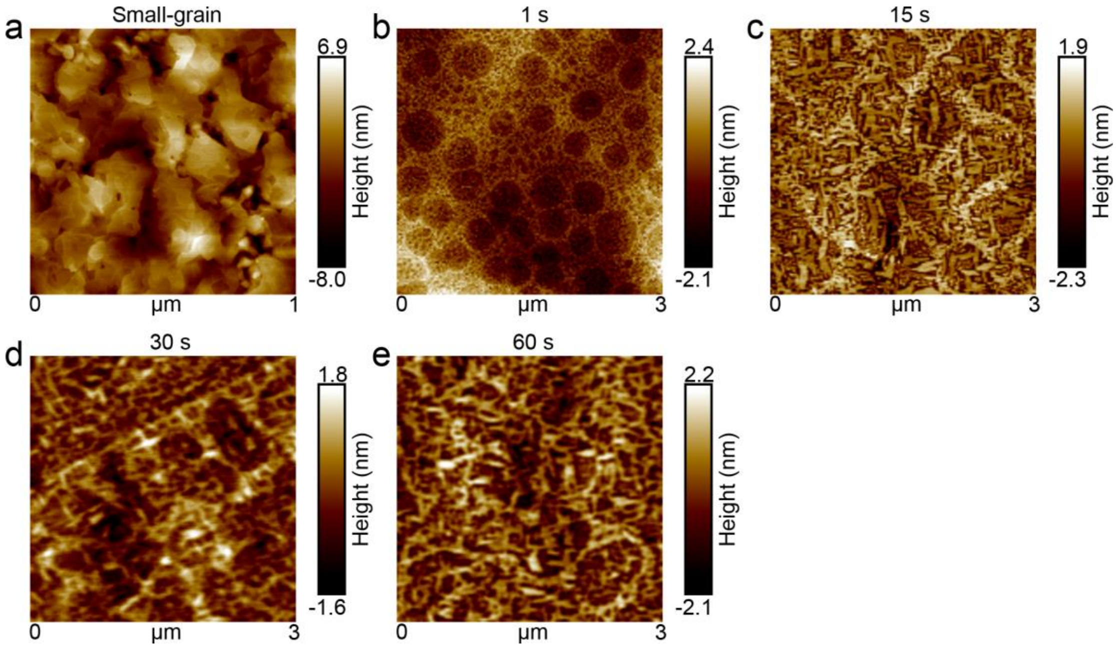


**Supplementary Figure 7| AFM images.** (**a**) the small-grain film, and different large-grain films with different degassing time, (**b**) 1 s. (**c**)15 s. (**d**) 30 s. (**e**) 60 s.

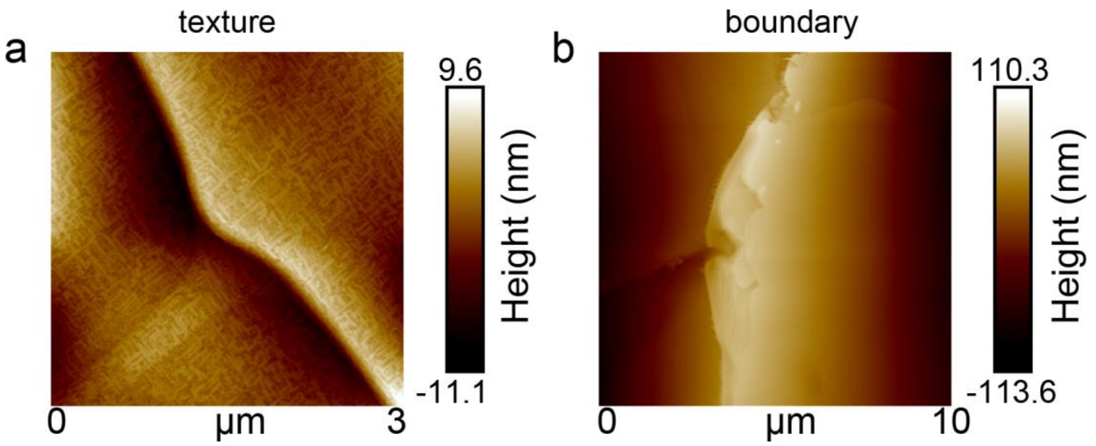


**Supplementary Figure 8| AFM images of the texture and the grain boundary for the large grain film (30 s).** (**a**) texture. (**b**) grain boundary.

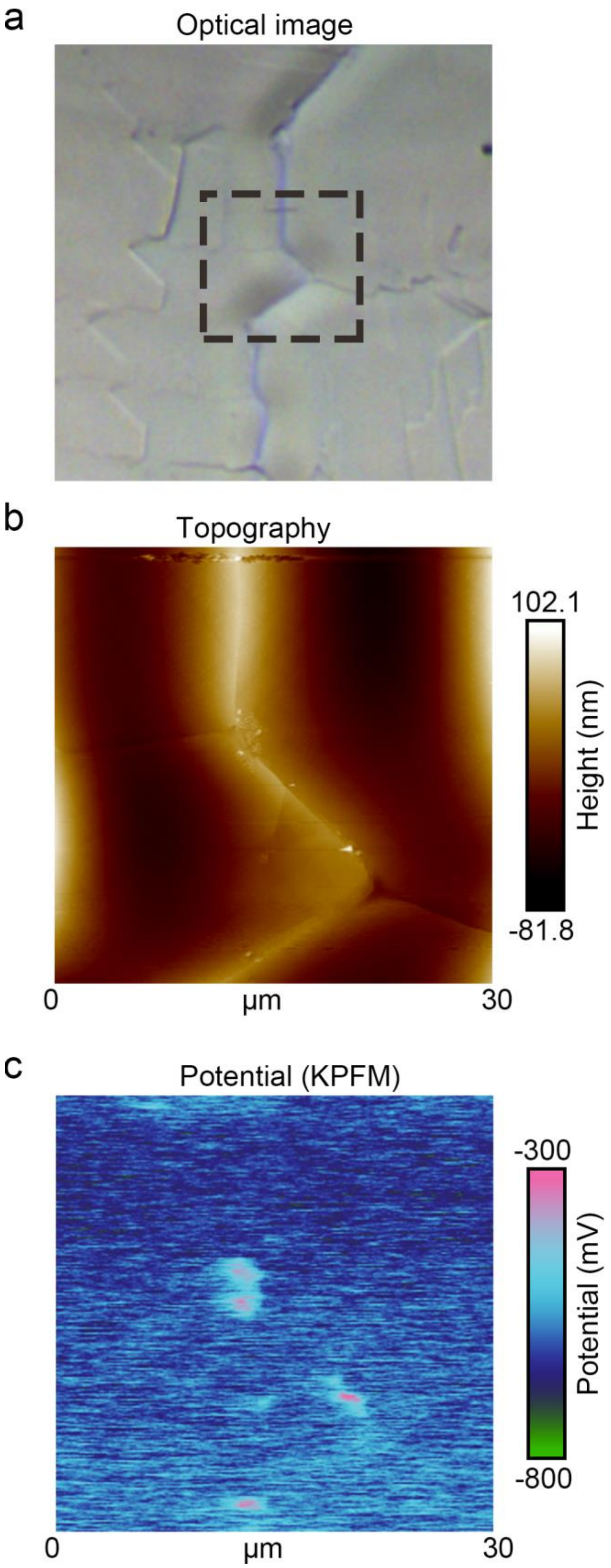


**Supplementary Figure 9| KPFM measurements.** (**a**) Optical image of the large-grain film; (**b**) AFM topographic image; (**c**) Potential image from the marked area in (**a**).

**Supplementary Note 2: The analysis of KPFM image**

Supplementary Figure 7 shows the optical, AFM and KPFM images of two adjacent grains in the large-grain film. As shown in Supplementary Figure 7 (b), the film is relatively smooth with low undulation, and Supplementary Figure 7 (c) shows the KPFM image of the same area in (b). The KPFM was acquired by a two-pass scan process, where the topography was obtained in the first scan and then the AFM tip was lifted up by 150 nm to measure the surface potential of the same line. It is clear that there is no significant difference in the potential distribution over the two adjacent grains including the grain boundary. Note: there are some impurities in the grain boundary, showing large difference in potential.

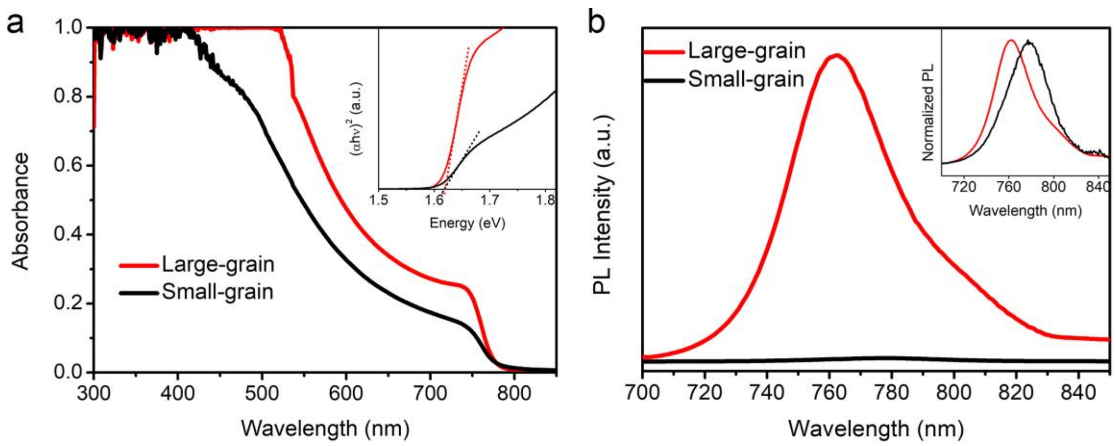


**Supplementary Figure 10| Optical properties of $MAPbI_3$ perovskite films.** (**a**) Measured UV-vis absorbance spectra of the small-grain and the large-grain perovskite films, the inset is the corresponding Tauc plots; (**b**) The corresponding PL spectra, which are normalized and shown in the inset.

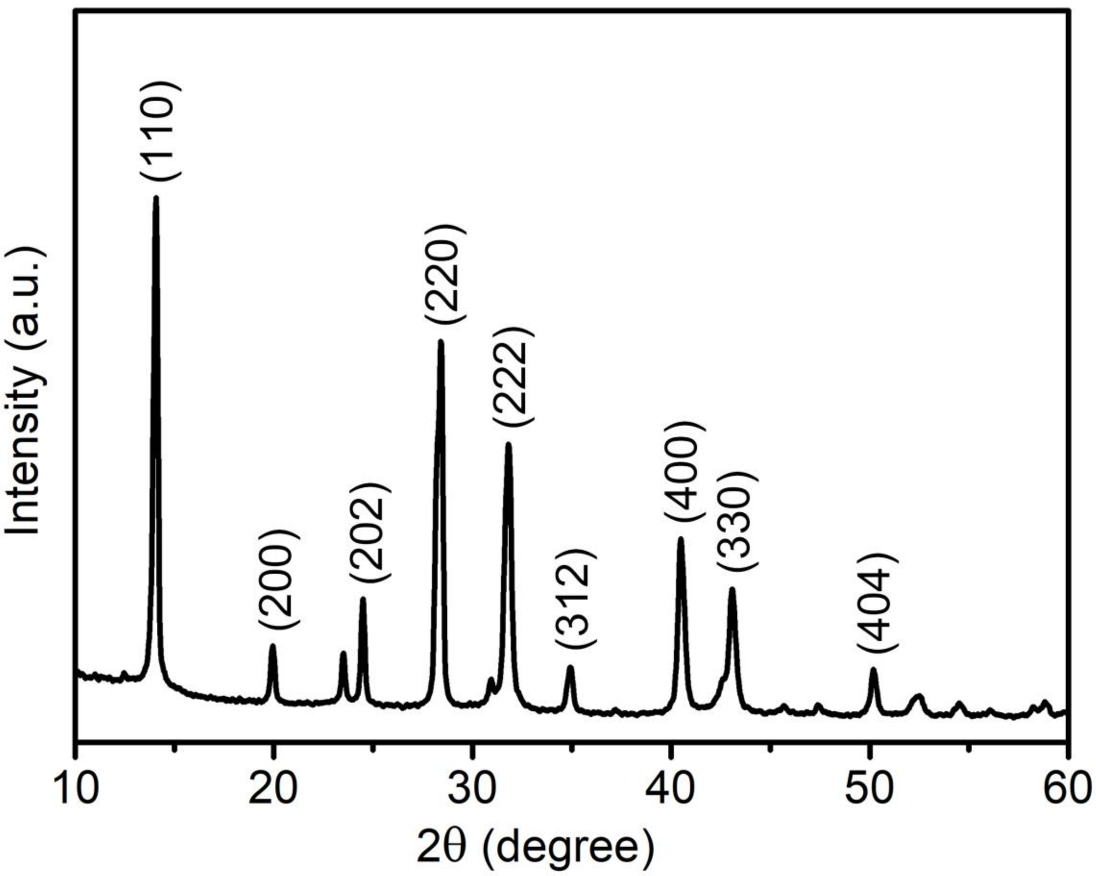


**Supplementary Figure 11| X-ray diffraction pattern of the grounded powder scraped from the large-grain $MAPbI_3$ film.**

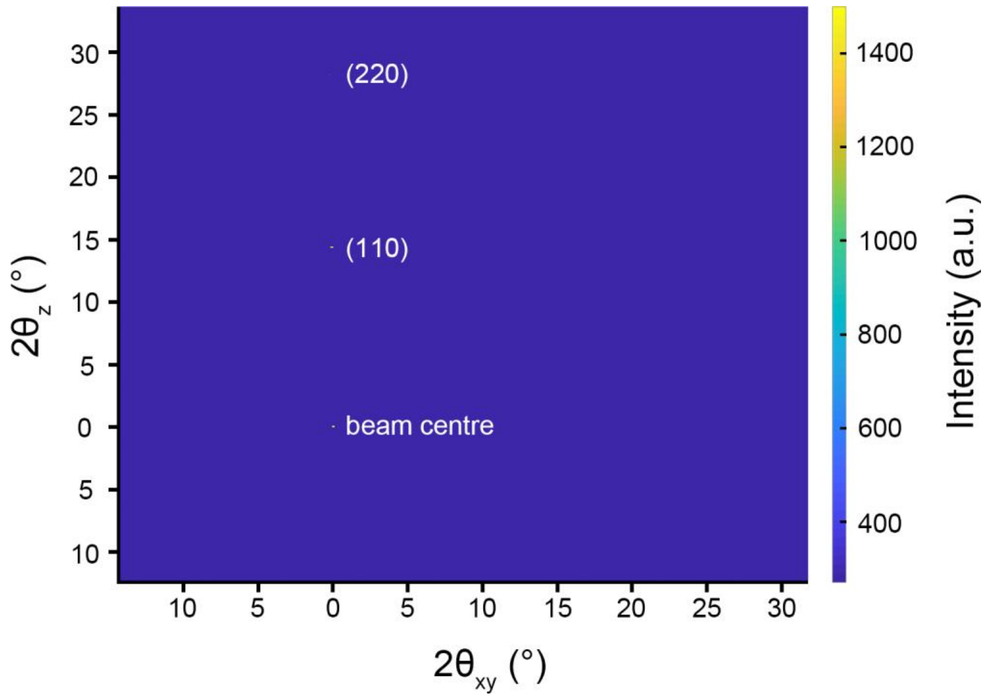


**Supplementary Figure 12| Two-dimensional image of X-ray diffraction.** Two-dimensional XRD image of one large grain from the large grain film. The image is made up of three photos taken at θ = 0, 7.05 and 14.1°.

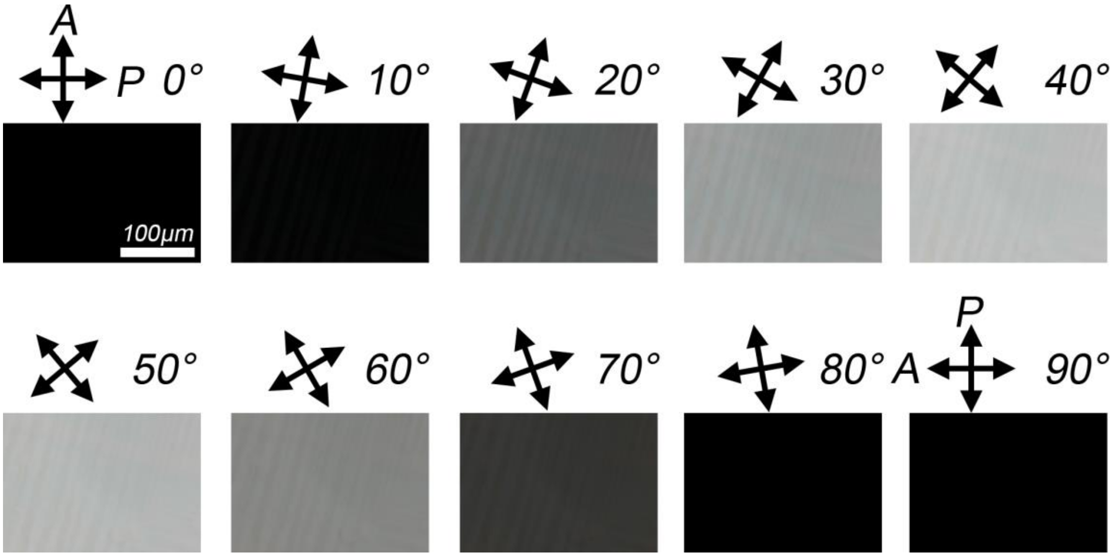


**Supplementary Figure 13| Optical micrographs of the large grain film viewed with cross polarizers, where the indicated angle is that of the polarizer relative to three O'clock in clockwise.**

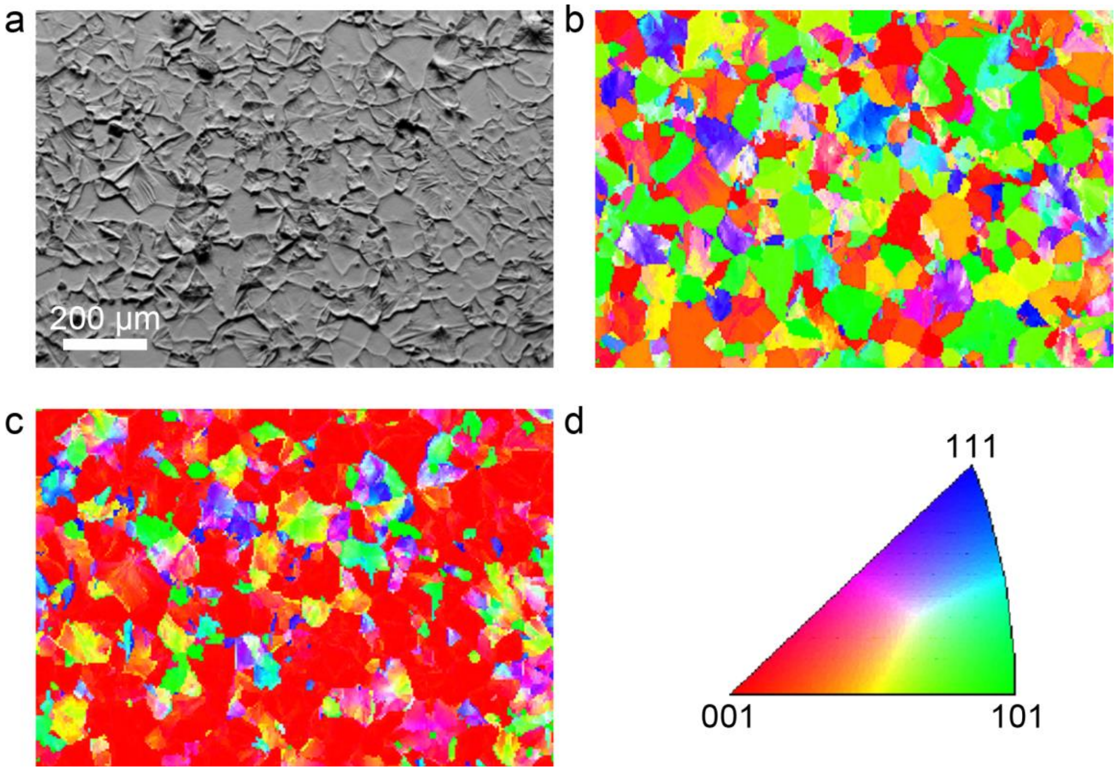


**Supplementary Figure 14| Electron backscatter diffraction (EBSD) of the $MAPbI_3$ film with tens of micron-sized grains.** (**a**) Backscattered electron (BSE) images. (**b**) X axis inverse pole figure (IPF) (parallel to the sample surface). (**c**) Z axis Inverse pole figure (IPF) (normal to the sample surface). (**d**) The standard color orientation reference system. The scale bar in (**a**) also applies to (**b**) and (**c**).

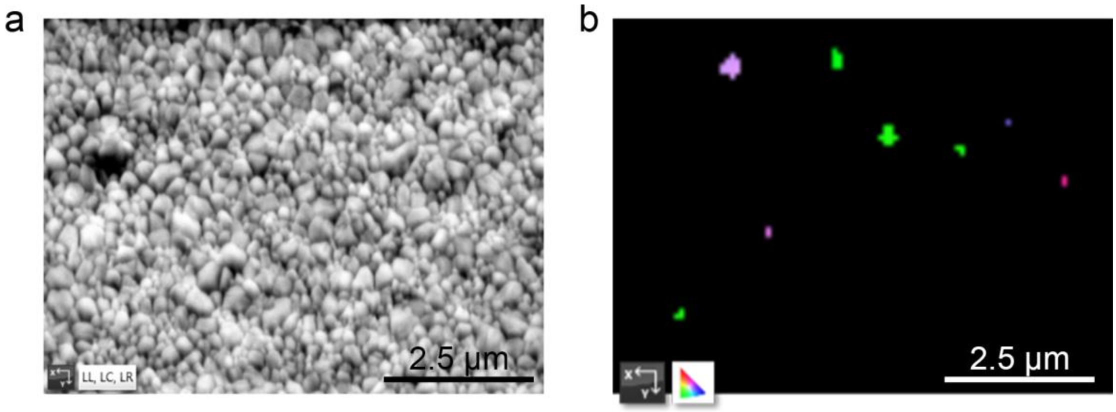


**Supplementary Figure 15| Electron backscatter diffraction (EBSD) of the small-grain film.** (**a**) Backscattered electron (BSE) image. (**b**) X axis inverse pole figure (IPF) (parallel to the sample surface).

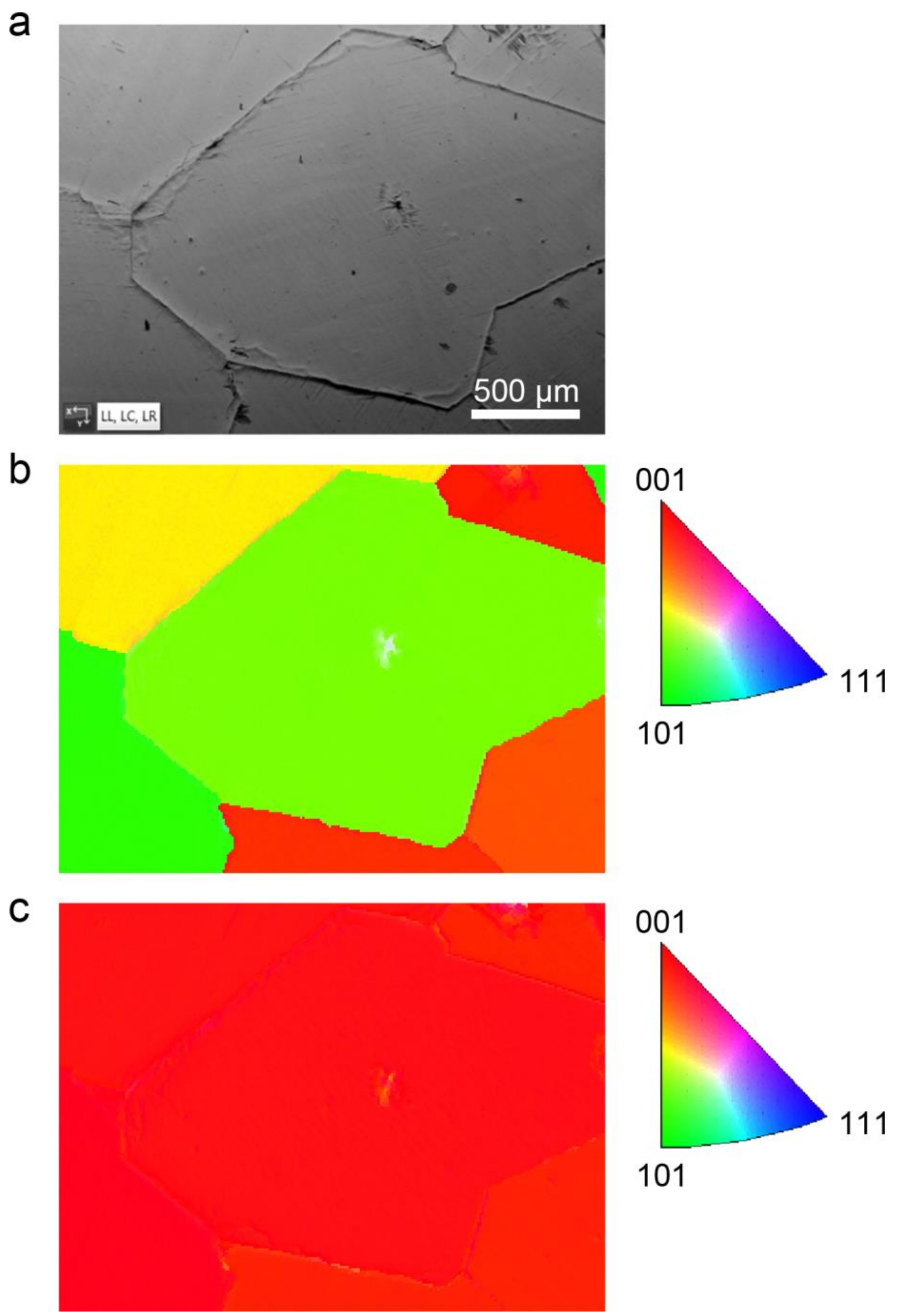


**Supplementary Figure 16| Electron backscatter diffraction (EBSD) of the large-grain $MAPbI_3$ film.** (**a**) Backscattered electron (BSE) image. (**b**) X axis inverse pole figure (IPF) (parallel to the sample surface). (**c**) Z axis inverse pole figure (normal to the sample surface). The scale bar in (**a**) also applies to (**b**) and (**c**).

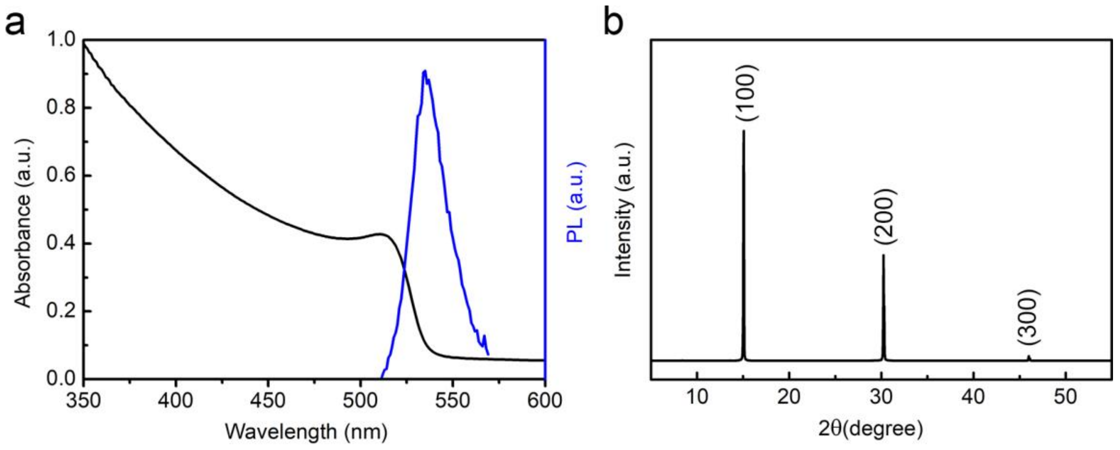


**Supplementary Figure 17| UV-Vis absorption, PL and XRD spectra of the large-grain $MAPbBr_3$ perovskite film.** (**a**) UV-Vis absorption and PL spectra. (**b**) XRD spectrum.

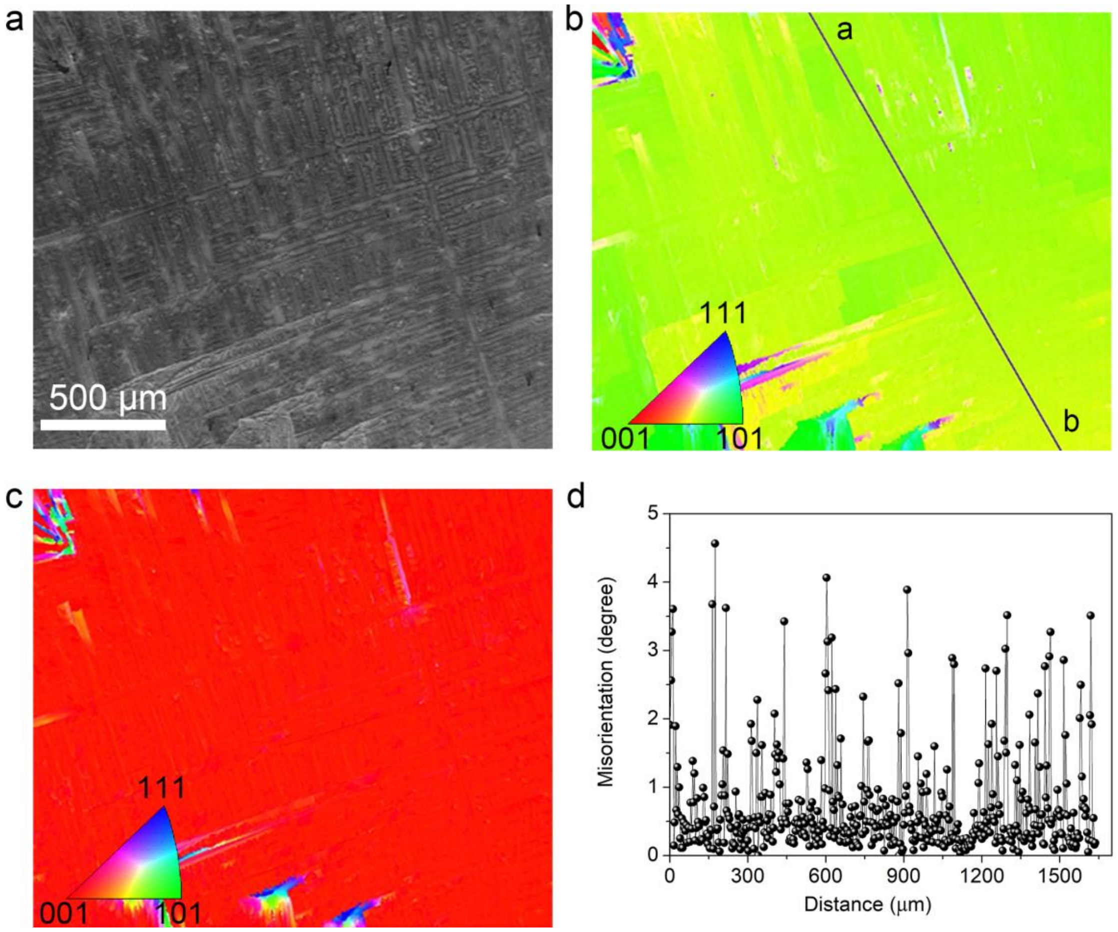


**Supplementary Figure 18| Electron backscatter diffraction (EBSD) of the large-grain $MAPbBr_3$ film.** (**a**) Backscattered electron (BSE) images. (**b**) X axis inverse pole figure (IPF) (parallel to the sample surface). (**c**) Z axis inverse pole figure (normal to the sample surface). (**d**) Point-to-point misorientation profile along the black line from a to b. The scale bar in (**a**) also applies to (**b**) and (**c**).

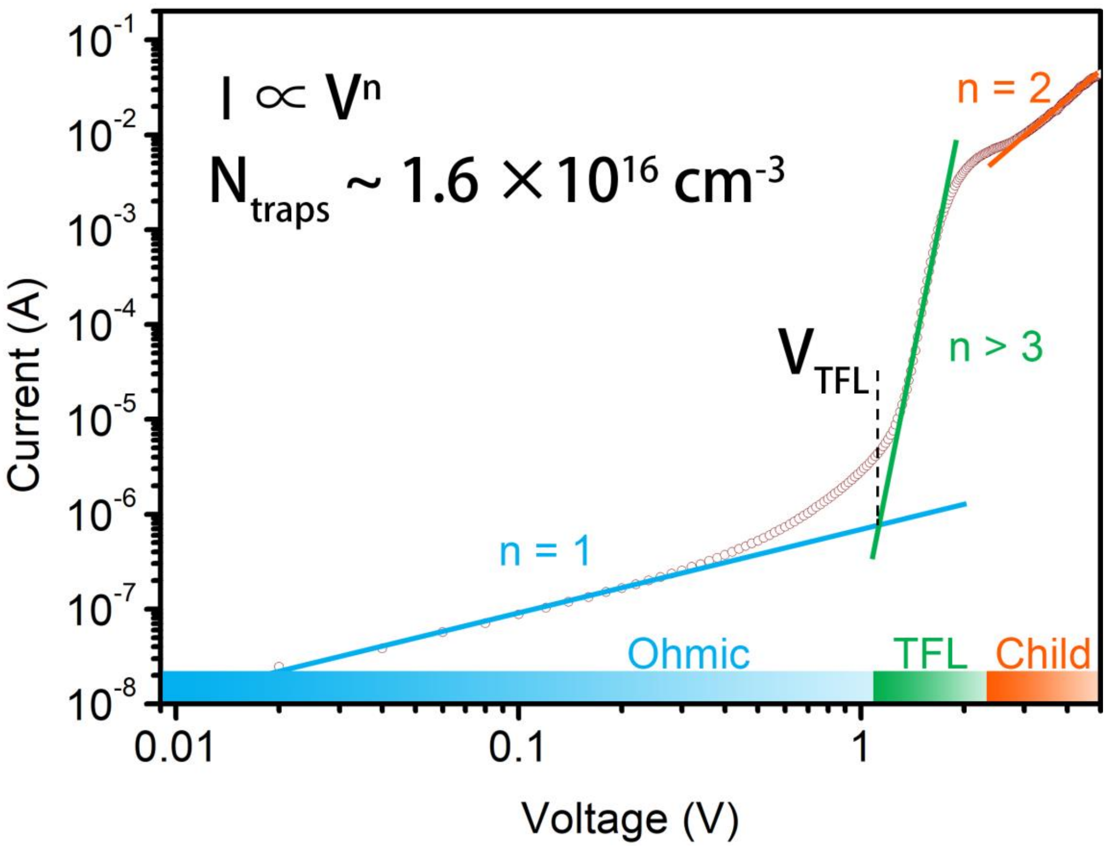


**Supplementary Figure 19| Current-voltage (*I-V*) traces**. Characteristic *I-V* trace of the small-grain perovskite film showing Ohmic, TFL and Child region. The onset voltage of the TFL region is ~ 1.25 V.

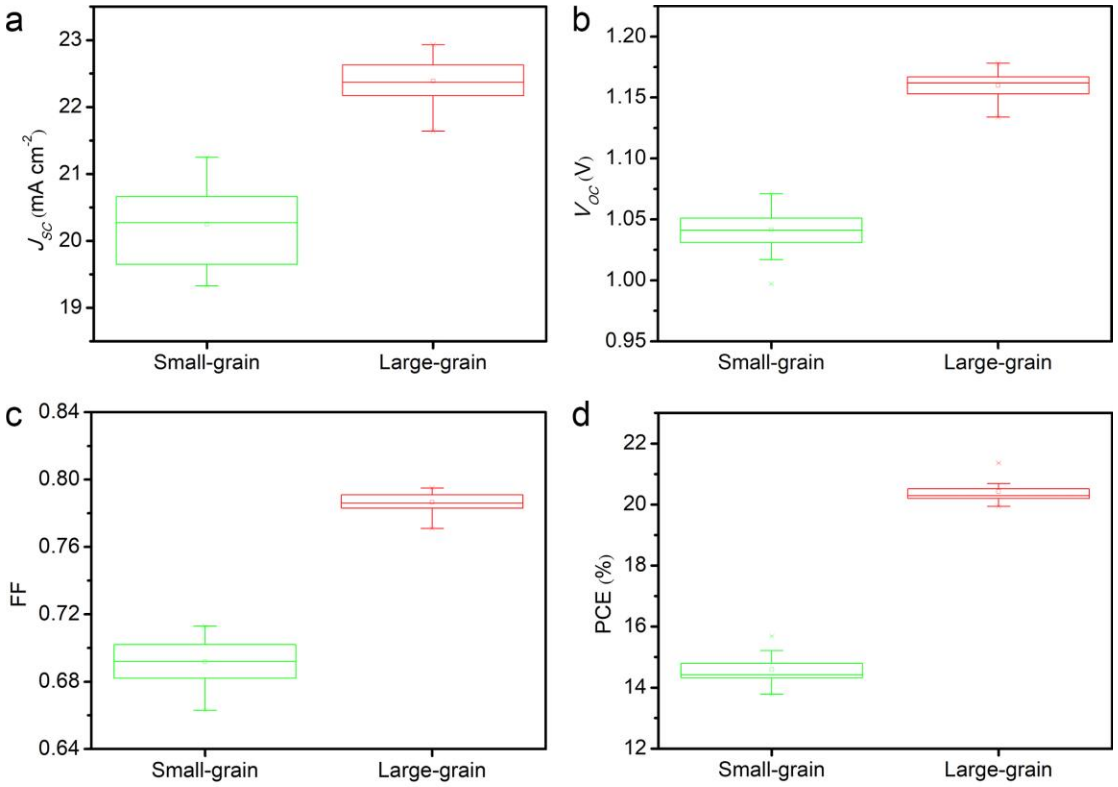


**Supplementary Figure 20| The comparison of the photovoltaic metrics for 20 devices fabricated with large-grain film and small-grain perovskite films.** (**a**) $J_{SC}$. (**b**) $V_{OC}$. (**c**) FF. (**d**) PCE. In the boxplots, the star represents the maximum and minimum values; the open square represents the mean value.

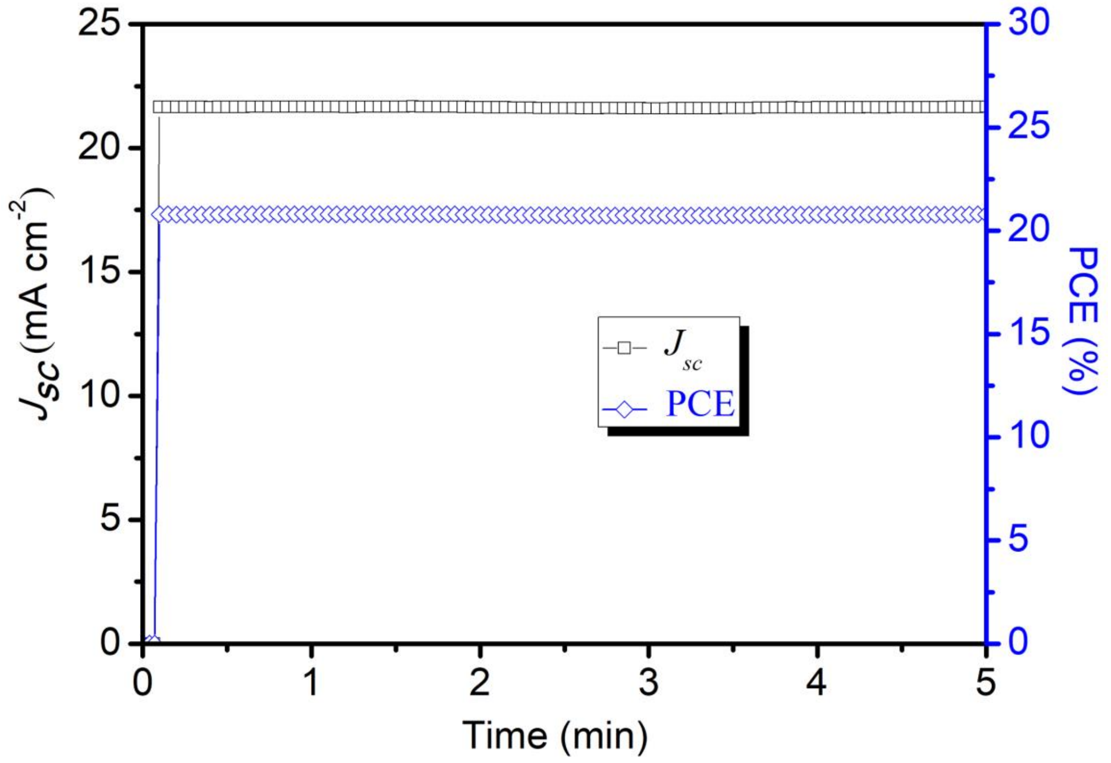


**Supplementary Figure 21| Stabilized maximum power output measurement of the large-grain device with bias at the maximum power point of 0.975 V.**

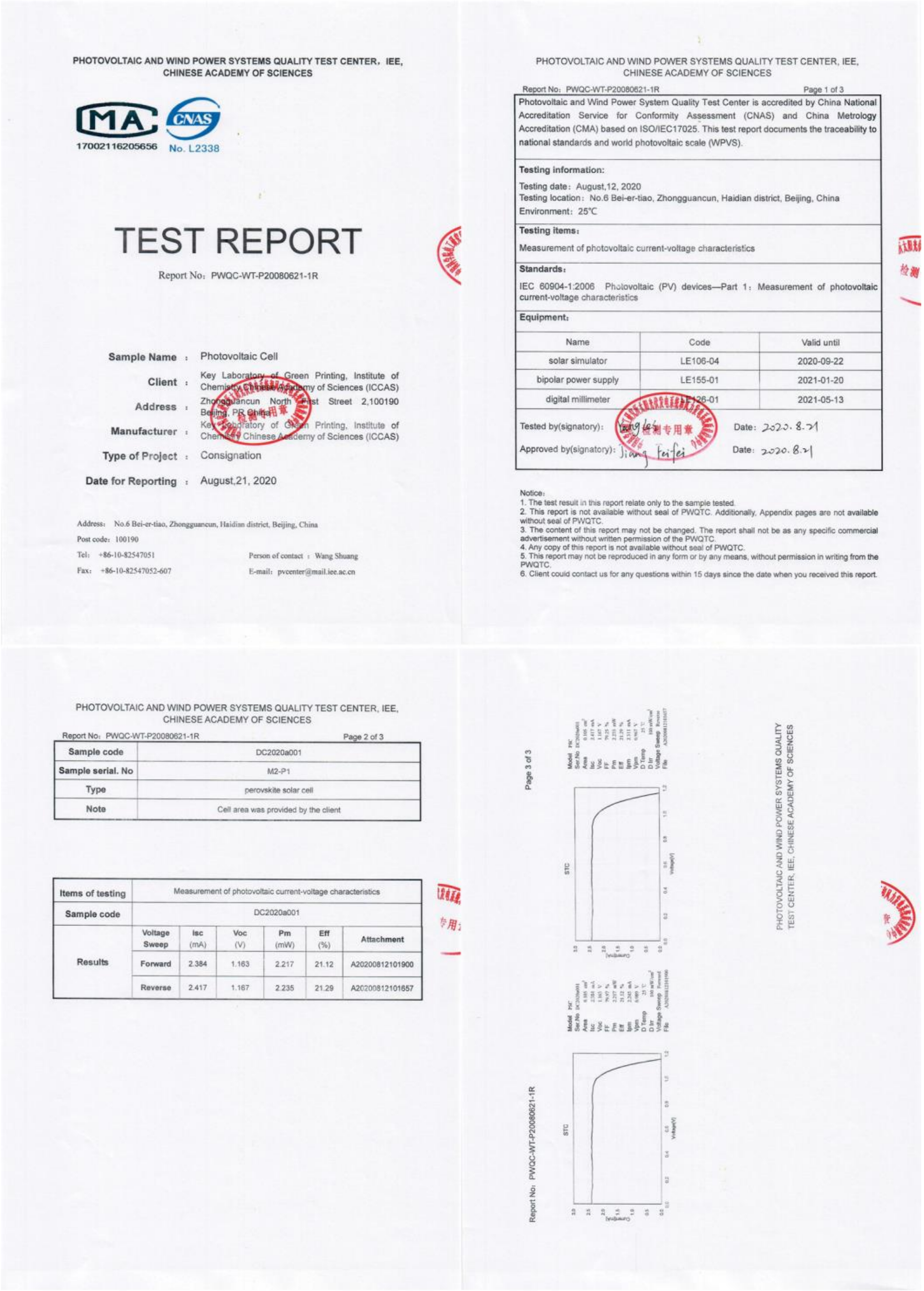

PHOTOVOLTAIC AND WIND POWER SYSTEMS QUALITY TEST CENTER，IEE，
CHINESE ACADEMY OF SCIENCES

170021162056 No. L2338

# TEST REPORT

Report No：PWQC-WT-P20080621-1R

| | |
|---|---|
| Sample Name： | Photovoltaic Cell |
| Client： | Key Laboratory of Green Printing, Institute of Chemistry Chinese Academy of Sciences (ICCAS) |
| Address： | Zhongguancun North First Street 2,100190 Beijing, PR China |
| Manufacturer： | Key Laboratory of Green Printing, Institute of Chemistry Chinese Academy of Sciences (ICCAS) |
| Type of Project： | Consignation |
| Date for Reporting： | August,21, 2020 |

Address： No.6 Bei-er-tiao, Zhongguancun, Haidian district, Beijing, China
Post code： 100190
Tel： +86-10-82547051
Fax： +86-10-82547052-607
Person of contact： Wang Shuang
E-mail： pvcenter@mail.iee.ac.cn

PHOTOVOLTAIC AND WIND POWER SYSTEMS QUALITY TEST CENTER, IEE,
CHINESE ACADEMY OF SCIENCES

Report No：PWQC-WT-P20080621-1R Page 1 of 3

Photovoltaic and Wind Power System Quality Test Center is accredited by China National Accreditation Service for Conformity Assessment (CNAS) and China Metrology Accreditation (CMA) based on ISO/IEC17025. This test report documents the traceability to national standards and world photovoltaic scale (WPVS).

**Testing information:**
Testing date：August,12, 2020
Testing location：No.6 Bei-er-tiao, Zhongguancun, Haidian district, Beijing, China
Environment：25℃

**Testing items：**
Measurement of photovoltaic current-voltage characteristics

**Standards：**
IEC 60904-1:2006 Photovoltaic (PV) devices—Part 1：Measurement of photovoltaic current-voltage characteristics

**Equipment：**

| Name | Code | Valid until |
|---|---|---|
| solar simulator | LE106-04 | 2020-09-22 |
| bipolar power supply | LE155-01 | 2021-01-20 |
| digital millimeter | [illegible]126-01 | 2021-05-13 |

Tested by(signatory)： Yang [illegible] Date： 2020.8.21
Approved by(signatory)： Jiang Feifei Date： 2020.8.21

Notice：
1. The test result in this report relate only to the sample tested.
2. This report is not available without seal of PWQTC. Additionally, Appendix pages are not available without seal of PWQTC.
3. The content of this report may not be changed. The report shall not be as any specific commercial advertisement without written permission of the PWQTC.
4. Any copy of this report is not available without seal of PWQTC.
5. This report may not be reproduced in any form or by any means, without permission in writing from the PWQTC.
6. Client could contact us for any questions within 15 days since the date when you received this report.

PHOTOVOLTAIC AND WIND POWER SYSTEMS QUALITY TEST CENTER, IEE,
CHINESE ACADEMY OF SCIENCES

Report No：PWQC-WT-P20080621-1R Page 2 of 3

| | |
|---|---|
| Sample code | DC2020a001 |
| Sample serial. No | M2-P1 |
| Type | perovskite solar cell |
| Note | Cell area was provided by the client |

| Items of testing | Measurement of photovoltaic current-voltage characteristics | | | | | |
|---|---|---|---|---|---|---|
| Sample code | DC2020a001 | | | | | |
| Results | Voltage Sweep | Isc (mA) | Voc (V) | Pm (mW) | Eff (%) | Attachment |
| | Forward | 2.384 | 1.163 | 2.217 | 21.12 | A20200812101900 |
| | Reverse | 2.417 | 1.167 | 2.235 | 21.29 | A20200812101657 |

Page 3 of 3

Report No：PWQC-WT-P20080621-1R

PHOTOVOLTAIC AND WIND POWER SYSTEMS QUALITY TEST CENTER, IEE, CHINESE ACADEMY OF SCIENCES

**Supplementary Figure 22| Certification document of the large-grain based perovskite solar cell (PHOTOVOLTAIC AND WIND POWER SYSTEMS QUALITY TEST CENTER CHINESE ACADEMY OF SCIENCES).** The device was measured with forward scanning from 0 V to -1.2 V and reverse scanning from -1.2 V to 0 V at 100 mV $s^{-1}$. For the forward scanning, $J_{SC}$, $V_{OC}$ and FF are 22.70 mA $cm^{-2}$, 1.16 V and 79.97, respectively, corresponding a PCE of 21.12%, while for the reverse scanning, $J_{SC}$, $V_{OC}$ and FF are 23.02 mA $cm^{-2}$, 1.17 V and 79.25, respectively, corresponding a PCE of 21.29%. The metal mask was used with an area of 0.105 $cm^2$.

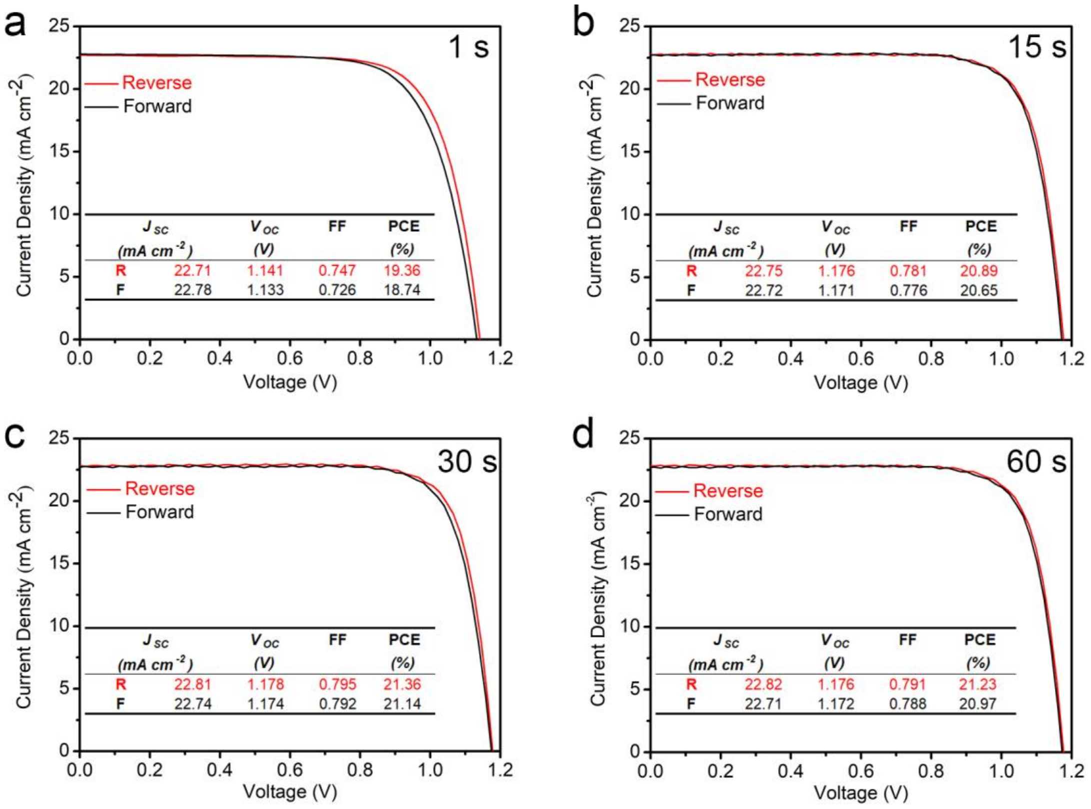


**Supplementary Figure 23| The representative *I-V* curves for the devices with the perovskite films crystallized from the liquid phase with a different time.** (**a**) 1 s, (**b**)15 s, (**c**)30 s, and (**d**) 60 s.

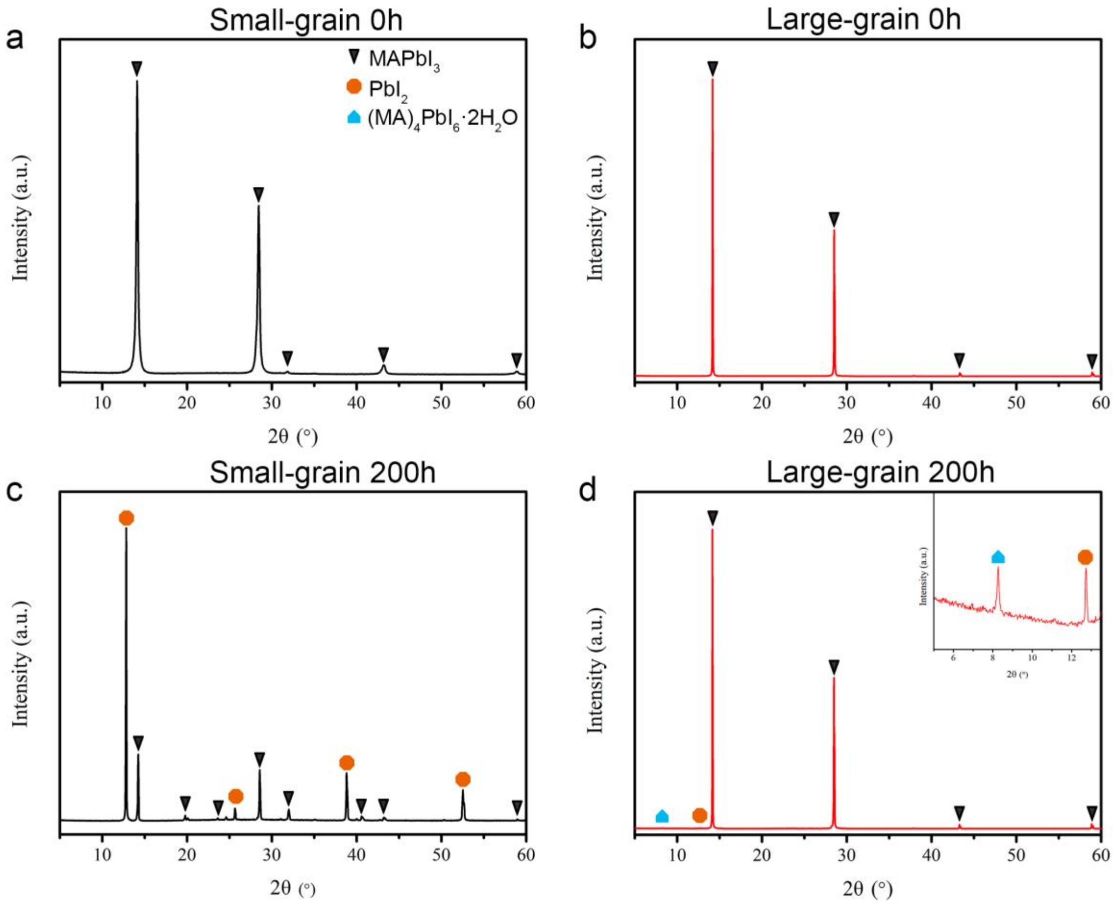


**Supplementary Figure 24| XRD patterns of fresh and ageing films.** (**a**) Fresh small-grain film. (**b**) Fresh large grain film. (**c**) The small-grain film stored at 30 °C, 65% RH in the dark for 200 h. (**d**) The large-grain film stored at 30 °C, 65% RH in the dark for 200 h. The inserted image is a partial enlargement of the original image.

**Supplementary Table 1| Intervals of the partial pressure of MA gas at the different temperatures for the crystallization of the $MAPbI_3$.**

| T/K | 373 | 393 | 413 | 433 | 453 |
|---|---|---|---|---|---|
| P/MPa | 0.058 ~ 0.039 | 0.144 ~0.096 | 0.198 ~ 0.168 | 0.312 ~ 0.279 | 0.512 ~ 0.444 |
