# Supplementary material for: Methylamine-assisted growth of uniaxial-oriented perovskite thin films with millimeter-sized grains
Source: Nat Commun. 2020 Nov 6;11:5402. doi: 10.1038/s41467-020-19199-6 (PMC7648077; doi:10.1038/s41467-020-19199-6)
Supplement: Supplementary file 2 — Reporting Summary [file 41467_2020_19199_MOESM2_ESM.pdf]

nature research

Corresponding author(s): Ke-Jian Jiang

Solar Cells Reporting Summary

Nature Research wishes to improve the reproducibility of the work that we publish. This form is intended for publication with all accepted papers reporting the characterization of photovoltaic devices and provides structure for consistency and transparency in reporting. Some list items might not apply to an individual manuscript, but all fields must be completed for clarity.

For further information on Nature Research policies, including our data availability policy, see Authors & Referees.

Experimental design

Please check: are the following details reported in the manuscript?

1. Dimensions

Area of the tested solar cells	☒ Yes	7.25 mm2 (methods)
	☐ No	*Explain why this information is not reported/not relevant.*
Method used to determine the device area	☒ Yes	Defined by the aperture.
	☐ No	*Explain why this information is not reported/not relevant.*

2. Current-voltage characterization

Current density-voltage (J-V) plots in both forward and backward direction	☒ Yes	Figure 4c
	☐ No	*Explain why this information is not reported/not relevant.*
Voltage scan conditions<br>*For instance: scan direction, speed, dwell times*	☒ Yes	J-V curves were measured with a scanning rate of 10 mV/s (voltage step of 20 mV and delay time of 100 ms)
	☐ No	*Explain why this information is not reported/not relevant.*
Test environment<br>*For instance: characterization temperature, in air or in glove box*	☒ Yes	All device are measured in air at room temperature.
	☐ No	*Explain why this information is not reported/not relevant.*
Protocol for preconditioning of the device before its characterization	☐ Yes	*State where this information can be found in the text.*
	☒ No	No preconditioning is required before characterization.
Stability of the J-V characteristic<br>*Verified with time evolution of the maximum power point or with the photocurrent at maximum power point; see ref. 7 for details.*	☒ Yes	Supplementary Figure 19
	☐ No	*Explain why this information is not reported/not relevant.*

3. Hysteresis or any other unusual behaviour

Description of the unusual behaviour observed during the characterization	☒ Yes	Figure 4c
	☐ No	*Explain why this information is not reported/not relevant.*
Related experimental data	☒ Yes	Figure 4c
	☐ No	*Explain why this information is not reported/not relevant.*

4. Efficiency

External quantum efficiency (EQE) or incident photons to current efficiency (IPCE)	☒ Yes	Figure 4d
	☐ No	*Explain why this information is not reported/not relevant.*
A comparison between the integrated response under the standard reference spectrum and the response measure under the simulator	☒ Yes	Figure 4d
	☐ No	*Explain why this information is not reported/not relevant.*
For tandem solar cells, the bias illumination and bias voltage used for each subcell	☐ Yes	*State where this information can be found in the text.*
	☒ No	Single-layer solar cell

5. Calibration

Light source and reference cell or sensor used for the characterization

☒ Yes
☐ No

The light source was a 300 W collimated xenon lamp (Newport) calibrated with the light intensity to 100 mW cm-2 under AM 1.5G solar light conditions by a certified silicon solar cell.

Explain why this information is not reported/not relevant.

Confirmation that the reference cell was calibrated and certified

☒ Yes
☐ No

The light intensity was calibrated by reference solar cell by Enli Tech.

Explain why this information is not reported/not relevant.

Calculation of spectral mismatch between the reference cell and the devices under test

☒ Yes
☐ No

Mismatch factor of 1 was used in our measurements.

Explain why this information is not reported/not relevant.

6. Mask/aperture

Size of the mask/aperture used during testing

☒ Yes
☐ No

7.25 mm2

Explain why this information is not reported/not relevant.

Variation of the measured short-circuit current density with the mask/aperture area

☐ Yes
☒ No

State where this information can be found in the text.

Only measured the J-V curve at a fixed aperture

7. Performance certification

Identity of the independent certification laboratory that confirmed the photovoltaic performance

☒ Yes
☐ No

Supplementary Figure 20

Explain why this information is not reported/not relevant.

A copy of any certificate(s)
Provide in Supplementary Information

☒ Yes
☐ No

Supplementary Figure 20

Explain why this information is not reported/not relevant.

8. Statistics

Number of solar cells tested

☒ Yes
☐ No

20

Explain why this information is not reported/not relevant.

Statistical analysis of the device performance

☒ Yes
☐ No

Supplementary Figure 18

Explain why this information is not reported/not relevant.

9. Long-term stability analysis

Type of analysis, bias conditions and environmental conditions
For instance: illumination type, temperature, atmosphere humidity, encapsulation method, preconditioning temperature

☒ Yes
☐ No

Figure 5

Explain why this information is not reported/not relevant.
